# Maternal and neonatal outcomes of childbirth care in a Freestanding Birth Centre*

**DOI:** 10.1590/1518-8345.7208.4596

**Published:** 2025-07-28

**Authors:** Nathalie Leister, Gisele Almeida Lopes, Caroline de Oliveira Ferreira Iguchi, Thalita Vital Botelho, Maria Luiza Gonzalez Riesco

**Affiliations:** 1 Universidade de São Paulo, Escola de Enfermagem, São Paulo, SP, Brazil.; 2 City St George’s, University of London, School of Health and Medical Sciences, London, United Kingdom.; 3 Associação Comunitária Monte Azul, Casa Angela - Centro de Parto humanizado, São Paulo, SP, Brazil.

**Keywords:** Birthing Centers, Birth Setting, Midwifery, Health Care Outcome Assessment, Cross-Sectional Studies, Parturition

## Abstract

to compare maternal and neonatal care outcomes based on women’s parity and to describe neonatal morbidity and mortality among newborns of women admitted in labor.

a cross-sectional study involving 3,397 women admitted for childbirth at a Freestanding Birth Centre and their newborns. The exposure variable was parity, and the outcomes included the use of oxytocin and amniotomy, type of birth, perineal trauma, postpartum hemorrhage, maternal and neonatal transfer, and newborn admission to neonatal intensive or intermediate care units. Data were analyzed descriptively and through logistic regression.

primiparity was associated with a higher likelihood of receiving oxytocin and amniotomy, intrapartum transfer, second-degree tear, episiotomy, postpartum hemorrhage, cesarean section, forceps-assisted birth, and neonatal admission to neonatal intensive or intermediate care units. Births predominantly occurred in semi-seated and upright positions, either on a bed or in the birthing tub. The maternal transfer rate was 21.8%, while the neonatal transfer rate was 3.3%.

primiparity is a predictor of the analyzed interventions and unfavorable maternal and neonatal outcomes. However, the studied Freestanding Birth Centre can be considered a safe setting for childbirth among health pregnant women.

## Highlights

**(1)** Freestanding Birth Centers (FBC) are a safe option for the care of low-risk women and their newborns. **(2)** Primiparous women are more likely to receive oxytocin and amniotomy compared to multiparous women. **(3)** Primiparous women have a higher likelihood of intrapartum transfer than multiparous women. **(4)** Primiparous women are at a greater risk of perineal trauma than multiparous women. **(5)** Primiparous women have a higher chance of having forceps-assisted birth or cesarean section than multiparous women.

## Introduction

In Brazil, the model of childbirth and birth care is hospital-centered, interventionist, and obstetric-led, characterized by high cesarean section rates, which accounted for 58% of births in 2022^(1)^. In obstetric units, even in straightforward pregnancies, care is provided with routine interventions that lack clinical or obstetric justification, contrary to scientific literature recommendations and guidelines from the World Health Organization (WHO)^(2-3)^.

This model has been questioned in the country since the 1990s due to the excessive number of cesarean sections and the stagnation of maternal and perinatal mortality rates^(4)^. Since then, public policies have been developed to improve care within the Unified Health System (SUS). Among these policies, the introduction of Birth Centers (BC) in 1999 and the *Rede Cegonha* (Stork Network) policy in 2011 (recently updated by *Rede Alyne*) stand out. This initiative introduced a set of changes aimed at ensuring high-quality care, providing more suitable birth settings based on the pregnant woman’s choice, having midwives and nurse-midwives responsible for care^(5)^.

Descriptive and observational studies conducted in Freestanding Birth Centers (FBC) in Brazil - also known as Birth Houses - indicate the implementation of best practices. These include respecting the right to a birth companion throughout the whole stay, admitting women in the active phase of labor, utilizing non-pharmacological pain relief methods, allowing free choice of birth position, and ensuring the judicious use of procedures such as amniotomy, oxytocin administration, and episiotomy^(6-8)^.

Additionally, FBC promote autonomy and satisfaction among those receiving care^(7,9)^. Given the positive outcomes, even when maternal or neonatal transfers to obstetric units occur, the demand for out-of-hospital birth settings has been growing internationally^(10)^.

The favorable results observed in FBC depend on proper eligibility criteria and screening of straightforward pregnancies^(11-12)^. Studies report positive outcomes regardless of parity, although primiparous women have higher rates of transfer, cesarean section, postpartum hemorrhage (PPH), blood transfusion, and suspected or confirmed chorioamnionitis. Similarly, their newborns have a higher likelihood of Apgar <7 at the 5^th^ minute, neonatal intensive or intermediate care unit (NICU) admission, sepsis, and neonatal death^(11,13-15)^.

Recognizing these favorable maternal and neonatal outcomes, the Brazilian Ministry of Health launched the National Guideline for Normal Birth Care in 2016. This guideline recommends that pregnant women receive evidence-based and accessible information about the risks and benefits associated with different birth settings^(16)^. From that point forward, further promotion of the model was expected, including the creation of new FBC and an increase in the number of births in these facilities. Strong evidence supports that these centers are safe, yield better maternal and perinatal outcomes, reduce cesarean section rates, improve childbirth satisfaction, and are economically more sustainable compared to straightforward pregnancy and birth care provided in obstetric units^(6,8-9)^.

It is important to highlight that the National Guideline were largely developed based on international studies and standards, while also aligning with Brazilian literature^(2,17)^. However, studies on the configuration, operation, care, and outcomes of FBC in Brazil remain scarce.

With the aim of contributing to and advancing knowledge on this model of care and disseminating maternal and neonatal outcomes, this study aims to: 1) Compare maternal and neonatal care outcomes based on the parity of women admitted in labor; 2) Describe neonatal morbidity and mortality data from a Brazilian FBC.

## Method

### Type of study

This is a cross-sectional study, reported in accordance with the Strengthening the Reporting of Observational Studies in Epidemiology (STROBE) guidelines for cross-sectional studies.

### Locus

The study was conducted at *Casa Angela* – Humanized Birth Center (FBC), located in the southern region of São Paulo, SP, Brazil. Detailed information about the study site is available on the institution’s website: https://www.casaangela.org.br/a-casa-angela.html.

**Figure 1 -:**
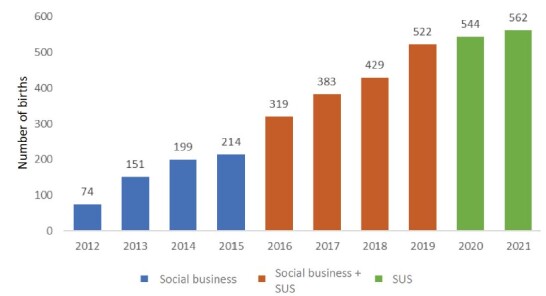
Distribution of the number of births by year and funding type (N = 3,397). São Paulo, SP, Brazil, 2012-2021

*Casa Angela* is managed by the non-governmental organization *Associação Comunitária Monte Azul*. It was founded in December 2009 and operated partially until 2011, offering prenatal care, childcare, and workshops for adolescents. In 2012, continuous care was implemented under a cross-subsidized social business model, where women from the local community who could not afford care were funded by those who could pay for services, along with donations and project-based funding. This model remained in place until the end of 2015, when a partnership with the Municipal Health Department of São Paulo was established to finance the provided care. Since 2020, all services have been fully funded by the Brazilian Unified Health System (SUS), with no direct costs to the users (Figure 1).

Patients at *Casa Angela* are either referred by a primary healthcare unit (UBS) or arrive spontaneously. Pregnant women undergo a risk screening process, and if they meet the eligibility criteria, they begin prenatal care at *Casa Angela* from 28 gestation weeks. Care is exclusively provided to healthy individuals who meet the criteria outlined in the Technical Manual for Birth Centers in the Municipality of São Paulo, which serves as the institution’s official protocol^(12)^. The clinical team consists of 20 midwives and obstetric nurses and nine nursing technicians

Admission for childbirth occurs after a second risk screening and reassessment of eligibility. Birth care is provided in a LBP room (Labor, Birth, and Postnatal), where the birthing person, newborn, and companions remain together from labor through the first two postpartum hours. After birth, the family is transferred to a private room for rooming-in, and discharge typically occurs around 24 hours postpartum. Two postpartum check-ups with a midwife or nurse-midwife are scheduled.

Non-pharmacological methods and Integrative and Complementary Health Practices (ICHP) are widely utilized. The model of care also ensures the continuous presence of up to two birth companions, immediate skin-to-skin contact for all newborns, and breastfeeding initiation within the first hour of life, unless emergency care is required. Although data on these practices are available, they were not included in this study, as they have already been reported in previous studies conducted at this site^(18-19)^.

Casa Angela has its own ambulance for immediate use in cases of intrapartum, postnatal, or neonatal transfers to its referral hospital: Campo Limpo Municipal Hospital *Dr. Fernando Mauro Pires da Rocha*, located 3 km away, with an approximate transport time of 10 minutes.

### Period

The study period was from 2012 to 2021 and included all records of pregnant women, their births, and maternal and neonatal transfers.

### Population and selection criteria

All pregnant women admitted for childbirth at the FBC during the study period were included (n=3,431). Pregnant women whose institutional records could not be located (n=5) and those who gave birth while in transit to the facility, arriving for postpartum care (n=29), were excluded. The final study population consisted of 3,397 women.

### Variables

The exposure variable was parity (primiparous women, who had never given birth before admission to birth, or multiparous women, who had previously given birth to one or more babies).

Outcome variables included: use of oxytocin during labor or birth (yes or no); amniotomy (yes or no); type of birth (non-instrumental vaginal, forceps, or cesarean); perineal condition (intact; perineal tear classified as first-degree, second-degree, third-degree, fourth-degree; or episiotomy); postpartum hemorrhage (PPH) (yes or no); maternal transfer to a hospital (yes or no, intrapartum or postnatal); neonatal transfer (yes or no); neonatal admission to an intensive care unit (yes or no) or intermediate care unit (yes or no).

Additional variables included: age (years old); skin color (white, brown, black, or Asian); education level (incomplete primary, complete primary, complete secondary, or higher education); marital status (lives or does not live with a partner); monthly income (≤1, >1≤3, >3≤6, or >6 times the minimum wage); funding type (social business model or SUS); birth position (semi-seated, all fours, seated, squatting, lateral, standing, or other); birth location (bed, birthing tub, floor, birthing stool, shower, or other); Apgar scores at the 1st and 5th minutes (0 to 10); and neonatal morbidity and mortality related to childbirth (description of the reason for intensive care unit or intermediate care unit admission).

### Information sources and data collection instrument

The data sources included institutional records and registry books documenting the booking, birth, and transfers from the FBC, containing records of all care provided during the study period. To collect the data, the authors developed a data collection instrument that included demographic, clinical, obstetric, and neonatal care variables, which was completed for each pregnant woman included in the study.

### Data collection

Data collection was conducted by three midwives and one trained nurse-midwife between June 2018 and March 2020. Due to the COVID-19 pandemic, data collection was interrupted between 2020 and 2021, resumed in February 2022, and concluded in November 2022.

### Data treatment and analysis

The data collected using the instruments were entered into a spreadsheet in Microsoft Excel and later transferred to the statistical analysis software R v4.3.2 for analysis.

Categorical variables were described using absolute and relative frequencies, while continuous variables were presented as means and standard deviations.

The chi-square test was used to analyze associations between exposure and categorical outcome variables, and when necessary, the chi-square test with Monte Carlo simulation was applied. Binary and multinomial logistic regressions were used to calculate the odds ratio (OR). The confidence level was set at 95%.

### Ethical aspects

The study was approved by the Research Ethics Committee of the School of Nursing at the University of São Paulo (CEP-EEUSP) – approval No. 2.026.648 (2017) – and authorized by the Scientific Committee of *Casa Angela*. Exemption from obtaining informed consent was granted since data were collected from the institution’s records, booking logs, birth records, and transfer logs.

## Results

The 3,397 pregnant women who had care at *Casa Angela* between 2012 and 2021 were mostly primiparous (72.2%), with an average age of 27.6 years old (SD=5.4). The majority were white (56.9%), had completed higher education (47.2%), were in a stable union (65.9%), and had a monthly household income between 1 and 3 times the minimum wage (49.8%). Deliveries predominantly occurred in the semi-seated position (30.9%), on a bed (38.2%), or in a birthing tub (24.7%). The sample size (n < 3,397) for skin color, education level, marital status, and family income was due to missing information in the study’s data sources, with data loss ranging from 17.5% (education level) to 23.5% (family income) (Table 1).

Since the characteristics of the women presented in Table 1 were not considered exposure variables, these variables were not stratified by parity.

Regarding the newborns, the mean Apgar score at 1 minute was 8.9 (SD=1.2; min=0; max=10), and at 5 minutes, it was 9.7 (SD=0.8; min=0; max=10), indicating good vitality at birth. A total of 95.3% (n=3,236) and 99.4% (n=3,375) had Apgar scores ≥ 7 at the 1st and 5th minutes, respectively (data not shown in table).

**Table 1-:** Characteristics of women admitted to *Casa Angela*. São Paulo, SP, Brazil, 2012-2021

**Characteristic**	**Mean (SD) Minimum–Maximum**
Age (years old) (N=3,397)	27.6 (5.4) – 14- 46
	N (%)
Parity	3,397 (100)
Primiparous	2,451 (72.2)
Multiparous	946 (27.8)
Skin color	2,796 (100)
White	1,591 (56.9)
Brown	785 (28.1)
Black	310 (11.1)
Asian	85 (3.0)
Indigenous	25 (0.9)
Schooling	2,854 (100)
Complete Higher Education	1,346 (47.2)
Complete High School	1,285 (45.0)
Complete Elementary School	162 (5.7)
Incomplete Elementary School	61 (2.1)
Marital status	2,830 (100)
Stable union	1,865 (65.9)
Not in a Stable union	965 (34.1)
Family income (minimum wages)*	2,610 (100)
≤ 1	268 (10.3)
1 ┤ 3	1,302 (49.8)
3 ┤ 6	688 (26.4)
> 6	352 (13.5)
Birth position ^†^	2,746 (100)
Semi-seated	851 (30.9)
All-fours	488 (17.8)
Seated	469 (17.1)
Squatting	437 (15.9)
Lateral	298 (10.9)
Standing up	110 (4.0)
Others ^‡^	93 (3.4)
Birthplace ^†‡^	2,746 (100)
Bed	1,048 (38.2)
Birthing Tub	679 (24.7)
Floor	448 (16.3)
Birthing Stool	428 (15.6)
Shower	107 (3.9)
Other ^§^	8 (0.3)
Outside the LBP ^||^ Room	28 (1.0)

*Corresponds to the minimum wage in the year the woman had care at *Casa Angela*, from 2012 to 2021, ranging from BRL 622.00 to BRL 1,100.00 during the period; ^†^Includes only women who gave birth at *Casa Angela*; ^‡^Supine, kneeling, “Elevated-Knee” position (one knee elevated and the other supported); ^§^Chair, armchair, straddle seat; ^||^Labor, Birth, and Postnatal

The results of care and maternal outcomes analyzed according to parity are presented in Table 2. The total maternal transfer rate (intrapartum and postnatal) was 21.8% (n=738).

**Table 2-:** Care and maternal outcomes according to the parity of women admitted to *Casa Angela*. São Paulo, SP, Brazil, 2012-2021

**Variable**	**Primiparous**	**Multiparous**	**Total**	**p-value**
	**N (%)**	**N (%)**	**N (%)**	
Oxytocin	2.451 (100)	946 (100)	3,397 (100)	<0.001*
Yes	156 (6.4)	18 (1.9)	174 (5.1)	
No	2,295 (93.6)	928 (98.1)	3,223 (94.9)	
Amniotomy	2,451 (100)	946 (100)	3,397 (100)	<0.001*
Yes	339 (13.8)	86 (9.1)	425 (12.5)	
No	2,112 (86.2)	860 (90.9)	2,972 (87.5)	
Type of birth	2,451 (100)	946 (100)	3,397 (100)	<0.001*
Non-instrumental vaginal	2,147 (87.6)	935 (98.8)	3,082 (90.8)	
Cesarean section	270 (11.0)	10 (1.1)	280 (8.2)	
Forceps-assisted birth	34 (1.4)	1 (0.1)	35 (1.0)	
Perineal condition ^†^	2,085 (100)	915 (100)	3,000 (100)	<0.001 ^‡^
Intact perineum	226 (10.8)	242 (26.4)	468 (15.6)	
First-degree tear ^§^	1,177 (56.6)	545 (59.6)	1,722 (57.4)	
Second-degree tear	591 (28.3)	125 (13.7)	716 (23.9)	
Third-degree tear	13 (0.6)	1 (0.1)	14 (0.5)	
Fourth-degree tear ^||^	-	1 (0.1)	1 (0.0)	
Episiotomy ^||^	78 (3.7)	1 (0.1)	79 (2.6)	
Postpartum hemorrhage ^¶^	1,853 (100)	893 (100)	2,746 (100)	0.023*
Yes	184 (9.9)	65 (7.3)	249 (9.1)	
No	1,669 (90.1)	828 (92.7)	2,497 (90.9)	
Transfer	2,451 (100)	946 (100)	3,397 (100)	
Intrapartum	598 (24.4)	53 (5.6)	651 (19.2)	<0.001*
Postnatal	66 (2.7)	21 (2.2)	87 (2.6)	0.092*

*Chi-square test; ^†^Includes partial data from hospital birth; ^‡^Monte Carlo simulation; ^§^Includes vulvar lacerations; ^||^Hospital birth; ^¶^Includes data only from women who gave birth at *Casa Angela*

Among the women who experienced PPH, 58 (23.3%) were transferred to the hospital due to vital sign alterations or severe anemia (hemoglobin levels below 7.0 g/dL). All women who had cervical laceration (n=6; 0.2%) and 8 out of 14 women who had a third-degree perineal tear at *Casa Angela* were postnatally transferred to the hospital for specialized care (data not shown in table).

The neonatal transfer rate was 3.3% (n=113), with 3.3% (n=81) of newborns from primiparous women and 3.4% (n=32) of newborns from multiparous women (p=0.910, chi-square test) (data not shown in table).

Among the newborns transferred to the hospital, 88 (77.9%) were admitted to the rooming-in room for phototherapy, evaluation, or diagnostic testing, being discharged from the hospital or returning to *Casa Angela* after care (data not shown in table).

Out of the 3,397 women, 84 (2.5% or 25/1,000) had a newborn admitted to the NICU, with a statistically significant difference concerning parity, as shown in Table 3. The same table describes the indications for neonatal intensive or intermediate care unit admission. Indications for additional testing and routine hospital procedures include newborns with suspected malformations or conditions requiring further clarification; in general, these procedures follow the specific neonatology service protocol for each case.

In addition to the morbidities described in Table 3, there were also 11 cases (0.40%) of clavicle fracture and 2 cases (0.07%) of brachial plexus injury. There were 2 stillbirths (0.07%) and 2 early neonatal deaths (0.06%).

**Table 3-:** Newborn admission to Intensive* and Intermediate Care Unit^†^and its indications (N = 3,397) São Paulo, SP, Brazil, 2012-2021

Variable	Primiparous	Multiparous	Total	p-value
	**N=78 (%)**	**N=6 (%)**	**N=84 (%)**	
No NICU* admission	2,373 (69.85) ^‡^	940 (27.67) ^‡^	3,313 (97.5)	<0.001 ^§^
Intensive care unit admission*	53 (1.56) ^‡^	5 (0.15) ^‡^	58 (1.7)	
Intermediate care unit admission ^†^	25 (0.74) ^‡^	1 (0.03) ^‡^	26 (0.8)	
Indications for any NICU* admission			84 (100%)	
Respiratory distress			24 (28.6)	
Infection or infection risk/Antibiotic therapy			18 (21.4)	
Asphyxia/Perinatal hypoxia			10 (11.9)	
Low Apgar score			9 (10.7)	
Additional diagnostic testing			9 (10.7)	
Meconium aspiration syndrome			8 (9.5)	
Jaundice			4 (4.8)	
Seizure due to encephalopathy			1 (1.2)	
Routine hospitalization			1 (1.2)	

*Neonatal Intensive Care Unit; ^†^Intermediate Care Unit; ^‡^Due to the low percentage, values are presented with two decimal places; ^§^Chi-square test

Maternal outcomes that showed statistically significant differences were analyzed using regression models, with the calculation of OR and 95% CI (Table 4).

Among primiparous women, the likelihood of receiving oxytocin and amniotomy was 3.5 and 1.6 times higher, respectively, compared to multiparous women. The likelihood of experiencing second-degree tear, PPH, and intrapartum transfer was also higher among primiparous women (2.6, 40.4, and 5.5 times, respectively). For cesarean section, forceps-assisted birth, and episiotomy, the likelihood was also higher among primiparous women (11.8, 14.8, and 43.7 times, respectively), despite a wide 95% CI (Table 4).

Regarding newborn admission to NICU, results showed that the likelihood of neonatal admission to these units was 5.2 times higher among primiparous women (Table 4).

**Table 4-:** Odds ratio (OR) and 95% Confidence Interval (95% CI) for maternal outcomes and neonatal admission according to the parity of women admitted for childbirth at *Casa Angela*. São Paulo, SP, Brazil, 2012-2021

**Outcome (n=3,397)**	**OR (CI 95%)**	**p-value**
Oxytocin	3.5 (2.2-5.9)	<0.001*
Amniotomy	1.6 (1.3-2.1)	<0.001*
Cesarean section	11.8 (6.2-22.2)	<0.001 ^†^
Forceps-assisted birth	14.8 (2.0-108.3)	<0.008 ^†^
Second-degree perineal tear	2.6 (2.1-3.8) ^‡^	<0.001 ^†^
Episiotomy	43.7 (6.1-314.2) ^‡^	<0.001 ^†^
Postpartum hemorrhage	40.4 (1.1-1.9)	0.024*
Intrapartum transfer	5.5 (4.1-7.4)	<0.001 ^†^
Newborn admission to NICU ^§^	5.2 (2.4-13.3)	<0.001*

*Binary logistic regression; ^†^Multinomial logistic regression; ^‡^Calculated against intact perineum and first-degree perineal tear combined; ^§^Neonatal Intensive Care Unit/Neonatal Intermediate Care Unit

## Discussion

This study presents the results of the childbirth care process within the FBC model of care, based on the characterization of *Casa Angela*, the population served, the care provided, and maternal and neonatal outcomes over 10 years of operation. Given the scarcity and underutilization of FBC in Brazil, these results aim to contribute to the dissemination, expansion, promotion, and establishment of new FBC.

Available data on the characteristics of women receiving care differ both nationally and internationally, which makes direct comparisons challenging. Regarding skin color, unlike the findings of this study, in birth centers in Minas Gerais and Rio de Janeiro, the majority of women self-identified as black or brown^(6,8)^. However, studies conducted in the United Kingdom and the United States of America reported that over 88% of women identified as white British, white European, or any other white ethnicity^(14,20-21)^.

The profile of *Casa Angela* users, in terms of skin color, marital status, and education level, suggests limited access for individuals in vulnerable situations, highlighting the need for policies and local initiatives to improve awareness and accessibility of the service.

Regarding parity, the data align with a recent study conducted in Rio de Janeiro^(6)^ and Australia^(22)^, which also found a higher prevalence of primiparous women. However, these findings differ from studies in the United Kingdom^(14,23)^. The “Birthplace” study, which analyzed maternal and neonatal outcomes among women with straightforward pregnancies, reported that primiparous women were a minority in FBC in the United Kingdom, and 36% of those who chose to give birth at birth centers were transferred to a hospital during labor^(15)^. This finding may discourage primiparous women from choosing these places as their first option for childbirth, as about one-third ultimately give birth in a hospital - a result that differs from this study.

At *Casa Angela*, various birthing positions were used, with a predominance of upright positions. This finding aligns with the care model of Brazilian FBC, which encourages and supports women’s choices. During the second stage of labor, supine positions (dorsal, gynecological, and lithotomy) should be avoided, as they are associated with a higher occurrence of fetal heart rate abnormalities and increased episiotomy rates, in addition to hindering spontaneous vaginal birth^(24)^. Recent FBC studies do not report the birthing positions chosen by women, possibly due to the unavailability of this information in institutional records or because positions are not commonly linked to morbidity and mortality outcomes, which are often the primary focus of studies.

Similarly, FBC encourage childbirth in locations other than the bed, which is frequently reported as the main birthing location. At *Casa Angela*, the second most common birthing location was the birthing tub. Waterbirth reduces the use of analgesics and anesthetics due to its relaxing effect, which helps relieve pain during contractions. Additionally, it decreases the practice of episiotomy by enabling the hands-off approach (birth occurring without the professional touching the woman’s perineal region), reduces the risk of PPH, increases the likelihood of an intact perineum, and enhances maternal satisfaction with birth care. Moreover, waterbirth does not affect any neonatal clinical outcomes for the newborn^(25)^.

Complications such as severe cervical or perineal tear are rare in birth centers. It is important to highlight that during the study period, no episiotomies were performed at *Casa Angela*, whereas rates between 1.0% and 8.3% have been reported in other FBC^(6,22-23)^. The non-episiotomy approach is supported by scientific evidence on non-episiotomy protocols, as this intervention does not prevent severe tear^(26)^.

Less interventionist practices influence perineal trauma outcomes. Thus, episiotomy rates are lower, and perineal integrity is higher in birth occurring at FBC^(19)^. The classification of tear severity may vary, but with team training, it can be improved^(27)^. At *Casa Angela*, perineal abrasions, vulvar injuries, and inner labial lacerations are classified as first-degree tear.

In general, third- and fourth-degree tear rates are low, but the data from this study differ from those found in a systematic review of high-income countries, where the rate was 2.7%^(28)^. This difference requires further investigation but may be related to the (un)availability of perineal data from women who were transferred intrapartum to the hospital or to underreporting.

No studies reporting cervical laceration incidence were found. At *Casa Angela*, severe cervical and perineal tear are reasons for postnatal transfer.

Regarding PPH, although it occurred in 9.1% of births in this study, only 2.1% of women required hospital transfer for this reason. International studies report similar data: in Australia, 3.4% of women who experienced PPH required postpartum transfusion^(22)^; in FBC located in high-income countries, blood loss exceeding 1,000 mL occurs in approximately 1.2% of births^(28)^.

Since medications for PPH prevention and management are part of clinical protocols^(12,16)^, cases without vital sign alterations or post-hemorrhagic anemia diagnosis can be treated and monitored by professionals at the birth center without the need for hospital transfer.

A study on risk factors for hospital admission due to PPH, defined as blood loss >1,000 mL, classified the risk as moderate for women with a uterine scar or more than three previous vaginal births^(29)^. No studies were found associating parity as an independent risk factor for PPH, particularly in FBC. However, this study found that being primiparous increases the likelihood of PPH. This finding may be related to the fact that antepartum risk factors for PPH render a pregnant woman ineligible for childbirth at the birth center. Thus, in FBC, intrapartum risk factors for PPH - such as prolonged labor, third- and fourth-degree perineal tear, and failure to progress - are more prevalent among primiparous women than multiparous women^(30)^.

Regarding type of birth it is known that high cesarean section rates are not beneficial and increase maternal morbidity and mortality, especially in low- and middle-income countries^(31)^. According to the WHO, cesarean section rates between 10% and 15% are considered optimal for ensuring positive surgical outcomes and experiences^(32)^. In this regard, the 58% cesarean section rate in Brazil^(1)^ should be critically analyzed, as it far exceeds the recommended threshold.

FBC were implemented within the Brazilian public health service not only to reduce cesarean section rates but also to improve care quality and provide humanized care, which respects women’s autonomy and the physiological birthing process^(5)^. Proper selection of straightforward pregnancies, well-trained and qualified professionals, and the FBC environment - which fosters physiological birth differently from hospital settings - can lead to fewer interventions without compromising maternal and neonatal outcomes^(33)^.

In this study, the spontaneous vaginal birth rate was 90.8%. A systematic review conducted in high-income countries reported a vaginal birth rate of 83% among women who began labor care in a FBC, compared to 61.7% among those who started labor care in a hospital^(28)^. An Australian study found that laboring in a FBC triples the likelihood of having a vaginal birth^(22)^. Similarly, an integrative review analyzing 56 studies showed that FBC births involve significantly fewer interventions^(7)^. Therefore, the choice of birth setting directly impacts the type of birth - planning childbirth outside the hospital environment increases the likelihood of vaginal birth and reduces cesarean and instrumental deliveries^(22,28)^.

The Normal Birth Care Guideline in Brazil and the Intrapartum Care Guideline in the United Kingdom recommend the judicious use of oxytocin and amniotomy^(16-17)^. The use of oxytocin in this study was even lower than in other Brazilian FBC, where oxytocin administration occurred in 27.5%-30.7% of cases^(6,8)^. However, the rate of amniotomy was higher than that reported in a FBC in Rio de Janeiro (1.2%)^(6)^. It was not possible to compare these data with studies conducted in high-income countries, as oxytocin is not available for use during labor in FBC - only for PPH prevention and treatment. When labor augmentation is required, the woman is transferred to the hospital^(17)^.

Intrapartum transfer rates vary widely in national and international studies (3.6%-37.4%)^(8,15,34-36)^, with higher rates among primiparous women (22.5%-34.1%) than multiparous women (2.9%-12.2%)^(22,34)^. This wide variation requires further investigation, as it may be associated with institutional differences, such as location, proximity to and agreements with the referral hospital, health care professionals, care and transfer protocols, availability of human and material resources, and other factors.

Regarding postnatal and neonatal transfers, there is less discrepancy among studies. Postnatal transfer rates are approximately 1.0%, while neonatal transfer rates range between 6.1% and 6.9%^(6,35)^.

The positive outcomes for women admitted for childbirth at *Casa Angela* are independent of parity - that is, maternal and neonatal interventions and outcomes indicate adherence to best practices for the success of the birth care model^(5,12,16,28)^. This conclusion is supported by national and international studies comparing straightforward pregnant women who planned childbirth at FBC versus those who planned hospital births^(8,15,21-22)^.

Regarding newborns, excellent Apgar scores were observed, aligning with those reported in various Brazilian FBC, where 95.2%-99.6% of babies had an Apgar score ≥ 7 at the 1^st^ minute and 98.9%-99.9% at the 5^th^ minute^(6,8)^. A recent U.S. study also found that 97.9% of newborns had an Apgar score ≥ 7 at the 5^th^ minute^(20)^. In FBC, factors associated with Apgar < 7 at the 5^th^ minute include shoulder dystocia, cord prolapse, intrapartum hemorrhage, and non-reassuring fetal status^(8)^ In comparative studies, Apgar scores in FBC births were similar to those observed in straightforward hospital births^(6,20)^.

Regarding neonatal transfer, resources in FBC are limited to straightforward birth care. Therefore, when medical evaluation, additional testing, or specific treatments such as phototherapy are required, the newborn must be transferred to the hospital. These reasons are not directly related to intrapartum care. As a result, in this study, the vast majority of newborns (77.9%) were transferred for non-labor-related reasons and admitted to for rooming-in, without serious clinical consequences or requiring NICU admission.

Regarding NICU admissions, similar findings have been reported in international studies (1.2%-2.6%), some without specifying intensive or intermediate care^(28)^. In this research, the main reasons for NICU admission were respiratory distress, infection or infection risk, low Apgar score, asphyxia or perinatal hypoxia, jaundice, and meconium aspiration syndrome.

Although we observed that the likelihood of NICU admission was 5.2 times higher among nulliparous women, a study on intra- and freestanding birth centers in the United Kingdom found no difference related to parity^(37)^.

Neonatal morbidity is one of the main concerns in childbirth care at FBC and highlights the importance of training midwives and obstetric nurses for the assessment and immediate care of newborn.

The same applies to neonatal mortality. This study found a rate of 0.06%, while a systematic review of high-income countries reported 0.02%^(28)^ and a U.S. study found 0.03%^(20)^. However, an Australian FBC study reported a 0.3% neonatal mortality rate^(36)^, five times higher than in this study.

It is important to emphasize that, despite the differences in morbidity and mortality rates among FBC studies, these rates are not different from those observed in newborns of low-risk women who gave birth in a hospital setting^(13)^.

Only two stillbirth cases (0.06%) occurred at *Casa Angela* during the period analyzed in this study, three times higher than the 0.02% stillbirth rate reported in the systematic review of high-income country studies^(28)^. However, an Australian study reported a stillbirth rate four times higher, at 0.24%^(22)^.

Mortality data should be analyzed cautiously due to the rarity of such events and the sample size of the studies. The rate of births occurring in FBC in Brazil is approximately 0.3%^(1)^, making it unfeasible to calculate a sample size with neonatal mortality as the primary outcome. Thus, when a death occurs in a study where the sample was not calculated for this outcome, the reported data may be biased.

Most studies focus on outcomes of births at FBC without following women and newborns after hospital transfers. This affects data quality and may be related to the ongoing tensions and challenges in the relationship between FBC and their referral hospitals.

Parity, as a predictor of interventions (oxytocin and amniotomy) and adverse outcomes (cesarean section, forceps-assisted birth, second-degree perineal tear, episiotomy, PPH, intrapartum transfer, and neonatal admission to NICU), indicates that primiparity increases the likelihood of these occurrences, except for postnatal transfer. Even though primiparous women had worse outcomes than multiparous women at this FBC, these outcomes were not worse than they would have been if they had given birth in a hospital^(6,8,21-22)^. This finding underscores the need for caution care of primiparous women compared to multiparous women but also reinforces the quality and safety of the care provided.

Key factors for birth care with fewer interventions and appropriate use of technology include the model of care, local culture, physical and material resources, work organization, human resources, leadership, and adaptability to change^(38-39)^. Despite the Brazilian Normal Birth Care Guideline recommending that healthcare professionals inform women about the safety of FBC for straightforward pregnancies, these facilities remain underutilized. This is due to the underuse of existing FBC, the lack of promotion for new FBC, and the failure to recognize the autonomy of midwives and nurse-midwives in birth care. It is worth noting that other countries also face similar barriers and challenges in expanding this model of care and facilities^(40-41)^.

This study aligns with the research priorities for nursing proposed by the Brazilian Nursing Association, which recommends analyzing care indicators in FBC^(42)^. However, given the positive outcomes observed in this work, further research on FBC should be encouraged. This birth setting, along with the leadership of midwives and nurse-midwives, plays a key role in challenging the prevailing biomedical and interventionist model of care in the country.

Since their inception and establishment, these facilities and professionals have aimed to offer and promote a biopsychosocial, physiological, and respectful model of care. We recommend that future studies include the outcomes of intrapartum, postnatal, and neonatal transfers, emphasizing that the FBC model of care is linked to a referral hospital. Not reporting these outcomes disregards the existence of this partnership, which enables the operation and sustainability of FBC^(39)^.

For the advance of scientific knowledge, we also recommend conducting prospective studies, despite the challenges and extended time required due to the limited number of births in these settings. In doing so, we contribute to expanding knowledge, enhancing the dissemination of care indicators, and promoting FBC as a safe birthing option for straightforward pregnancies.

This study’s limitations are primarily associated with its cross-sectional design, which relies on secondary data sources (institutional birth records and registry books) and may be subject to information and confounding biases. Over the years, certain data related to pregnancy person characteristics, birth location, and birthing position have undergone significant changes. As a result, it was not possible to collect complete information on skin color, education level, marital status, household income, birth position, and birth location for the entire study population - only for cases where this data was available in the sources.

## Conclusion

Compared to multiparous women, primiparous women have a higher likelihood of intrapartum transfer, oxytocin use, amniotomy, second-degree perineal tear, episiotomy, PPH, forceps-assisted birth, cesarean section, and neonatal admission to NICU.

However, even among primiparous women, care practices at the FBC reflect a limited use of interventions - oxytocin, amniotomy, and episiotomy -, a high prevalence of perineal integrity or mild perineal trauma (first-degree tear), and a preference for upright birthing positions and out-of-bed births. Notably, the low rates of cesarean section, forceps-assisted birth, and maternal and neonatal morbidity and mortality align with the low-risk profile of the women having care at this facility.

At the same time, hospital backup remains essential for both maternal and neonatal care, as transfers - though infrequent - are an expected part of the FBC model.

Thus, the findings reinforce the safety of childbirth at *Casa Angela* and the importance of FBC as a viable birthing option in Brazil, helping reduce high cesarean section rates and unnecessary interventions during labor.
