# Supplementary material for: Maternal and neonatal outcomes of childbirth care in a Freestanding Birth Centre
Source: Rev Lat Am Enfermagem. 2025 Jul 28;33:e4596. doi: 10.1590/1518-8345.7208.4596 (PMC12306936; doi:10.1590/1518-8345.7208.4596)
Supplement: Translation

# Translation: Resultados maternos e neonatais do atendimento ao parto em um Centro de Parto Normal peri-hospitalar*

**DOI:** 10.1590/1518-8345.7208.4597

**Authors:** Nathalie Leister, Gisele Almeida Lopes, Caroline de Oliveira Ferreira Iguchi, Thalita Vital Botelho, Maria Luiza Gonzalez Riesco

**Affiliations:** 1 Universidade de São Paulo, Escola de Enfermagem, São Paulo, SP, Brasil.; 2 City St George’s, University of London, School of Health and Medical Sciences, Londres, Reino Unido.; 3 Associação Comunitária Monte Azul, Casa Angela - Centro de Parto humanizado, São Paulo, SP, Brasil.

**Keywords:** Centros de Assistência à Gravidez e ao Parto, Local do Parto, Enfermagem Obstétrica, Avaliação de Resultados em Cuidados de Saúde, Estudos Transversais, Parto

## Abstract

comparar os desfechos assistenciais maternos e neonatais com a paridade das mulheres e descrever a morbimortalidade neonatal de bebês de mulheres que foram internadas em trabalho de parto.

estudo transversal com 3.397 mulheres admitidas para parto em Centro de Parto Normal peri-hospitalar e seus recém-nascidos. A variável de exposição foi paridade e os desfechos foram uso de ocitocina e amniotomia, tipo de parto, condição perineal, hemorragia pós-parto, transferências maternas e neonatais e internação do recém-nascido em unidades de cuidados intensivos ou intermediários (UTINeo/UCINeo). Os dados foram analisados descritivamente e por regressão logística.

a primiparidade associou-se a maior chance de receber ocitocina e amniotomia, ser transferida intraparto, ter laceração de 2º grau, episiotomia, hemorragia pós-parto, cesariana e fórcipe e ter o recém-nascido internado em UTINeo ou ICINeo. Os partos ocorreram, principalmente, nas posições semissentada e verticalizadas, na cama e na banheira. A taxa de transferência materna foi de 21,8% e a neonatal foi de 3,3%.

a primiparidade é preditora para as intervenções analisadas e para desfechos maternos e neonatais indesejáveis. No entanto, o Centro de Parto Normal peri-hospitalar estudado pode ser considerado seguro para mulheres de risco habitual parirem.

## Destaques

**(1)** O CPNp é seguro para o atendimento de mulheres de risco habitual e seus bebês. **(2)** Primíparas têm maior chance de receber ocitocina e amniotomia do que multíparas. **(3)** Primíparas têm maior chance de transferência intraparto do que multíparas. **(4)** Primíparas têm maior chance de trauma perineal do que multíparas. **(5)** Primíparas têm maior chance de parto com fórceps e cesariana do que multíparas.

## Introdução

No Brasil, o modelo de cuidado ao parto e nascimento é hospitalocêntrico, intervencionista e médico-centrado, caracterizado por altas taxas de cesariana, que em 2022 representaram 58% dos partos^(1)^. Em centros obstétricos, mesmo em gestações de risco habitual, a assistência geralmente é composta por intervenções rotineiras, não justificadas por razões clínicas ou obstétricas, contrariamente às recomendações científicas e da Organização Mundial da Saúde (OMS)^(2-3)^.

Este modelo tem sido questionado no país desde a década de 1990, em razão do excesso de cesarianas e da estagnação das taxas de mortalidade materna e perinatal^(4)^. Desde então, políticas públicas vêm sendo formuladas, visando a melhorias na assistência obstétrica no Sistema Único de Saúde (SUS). Entre estas, destacam-se a proposição dos Centros de Parto Normal (CPN), em 1999, e da política Rede Cegonha, em 2011 (recentemente atualizada pela Rede Alyne), que incluiu um conjunto de mudanças para promover uma assistência de qualidade, com oferta de locais mais adequados para dar à luz, de acordo com a escolha da gestante, e tendo obstetrizes e enfermeiras obstétricas como responsáveis pelo cuidado^(5)^.

Em estudos de caráter descritivo e observacional realizados em CPN peri-hospitalares (CPNp) brasileiros, também denominados Casa de Parto, os resultados apontam para o uso das boas práticas, como o cumprimento do direito ao acompanhante durante toda a internação, admissão em fase ativa de trabalho de parto, uso de métodos não farmacológicos para alívio da dor, adoção livre de posição para o parto e uso criterioso de práticas como amniotomia, administração de ocitocina e episiotomia^(6-8)^.

Além disso, os CPNp promovem a autonomia e a satisfação de quem recebe o cuidado^(7,9)^. Diante dos bons desfechos, mesmo quando há transferências maternas ou neonatais para centros obstétricos, a procura pelo parto em ambientes extra-hospitalares vem crescendo internacionalmente^(10)^.

Os bons resultados do atendimento prestado em CPNp dependem de critérios de elegibilidade e triagem adequados de gestantes de risco habitual^(11-12)^. Os estudos trazem resultados favoráveis independentemente da paridade, embora as primíparas tenham maiores taxas de transferência e de cesariana, hemorragia pós-parto ou necessidade de transfusão e infecção amniótica suspeita ou confirmada. Da mesma forma, seus bebês têm maior chance de Apgar < 7 no 5.º minuto, admissão em unidade de terapia intensiva neonatal, sepse e óbito neonatal^(11,13-15)^.

Reconhecendo os bons desfechos maternos e neonatais, o Ministério da Saúde brasileiro lançou, em 2016, as Diretrizes Nacionais de Assistência ao Parto Normal, que recomendam que as gestantes recebam informações baseadas em evidências científicas e em formato acessível, sobre os riscos e benefícios associados aos locais de parto^(16)^. A partir de então, esperava-se ainda mais incentivo ao modelo com a criação de novos CPNp e aumento do número de partos nestes locais, dado que há fortes evidências de que esses serviços são seguros, têm melhores resultados maternos e perinatais, reduzem as taxas de cesariana, melhoram a satisfação com o parto e são economicamente mais sustentáveis, quando comparados ao atendimento às gestações e partos de baixo risco em centros obstétricos^(6,8-9)^.

Vale destacar que as referidas Diretrizes Nacionais foram majoritariamente elaboradas com base em estudos e diretrizes internacionais, dialogando com a literatura brasileira^(2,17)^. Nesse sentido, percebe-se escassez de estudos sobre configuração, funcionamento, atendimento e desfechos nos CPNp no Brasil.

Com o intuito de contribuir e avançar no conhecimento sobre o modelo de cuidado e divulgar resultados maternos e neonatais da assistência prestada, os objetivos deste estudo são: 1) comparar os desfechos assistenciais maternos e neonatais com a paridade das mulheres que foram internadas em trabalho de parto; 2) descrever dados de morbimortalidade neonatal em um CPNp brasileiro.

## Método

### Tipo de estudo

Estudo observacional transversal analítico, com relato orientado pelo *Strengthening the Reporting of Observational Studies in Epidemiology* (STROBE) para estudos transversais.

### Local

O estudo foi realizado no CPNp Casa Angela - Centro de Parto Humanizado, localizado na zona sul da cidade de São Paulo, SP, Brasil. Informações detalhadas sobre o local do estudo podem ser obtidas no *site* da instituição (https://www.casaangela.org.br/a-casa-angela.html).

A Casa Angela é gerida pela organização não governamental Associação Comunitária Monte Azul. Foi fundada em dezembro de 2009 e funcionou parcialmente até 2011, com atendimentos de pré-natal, puericultura e promoção de oficinas para adolescentes. Em 2012, o atendimento foi implementado de modo ininterrupto, em um modelo de subsídio cruzado e negócio social, em que mulheres moradoras da região que não podiam pagar para terem atendimento eram financiadas pelo valor cobrado daquelas que podiam pagar pelo serviço, e também por doações e financiamentos de projetos desenvolvidos pela Casa. Esse modelo funcionou até o final de 2015, quando foi estabelecida uma parceria com a Secretaria Municipal de Saúde da cidade de São Paulo, para financiar o atendimento prestado. A partir de 2020, os atendimentos passaram a ser totalmente financiados pelo SUS, sem qualquer pagamento pelas usuárias (Figura 1).

**Figura 1 -:** Distribuição do número de atendimentos por ano de ocorrência e tipo de financiamento (N = 3.397). São Paulo, SP, Brasil, 2012-2021

As pessoas atendidas na Casa Angela são referenciadas pela unidade básica de saúde (UBS) ou chegam por demanda espontânea. As gestantes passam por uma triagem de risco e, se atenderem aos critérios de elegibilidade, iniciam o pré-natal na Casa a partir de 28 semanas de gestação. O atendimento é exclusivo para pessoas saudáveis que se enquadrem nos critérios constantes no Manual Técnico das Casas de Parto do Município de São Paulo, documento adotado como protocolo institucional^(12)^. A equipe clínica é composta por 20 obstetrizes e enfermeiras obstétricas e nove técnicas de enfermagem.

A admissão para o parto é feita após triagem de risco e reavaliação de elegibilidade. O atendimento ao parto é realizado no quarto PPP (pré-parto, parto e pós-parto), onde a parturiente/puérpera, o bebê e seus acompanhantes permanecem desde o trabalho de parto até 2 horas após o parto. No pós-parto, a família é encaminhada a um quarto privativo de alojamento conjunto (AC) e a alta ocorre em torno de 24 horas pós-parto. São marcadas duas consultas de puerpério com a obstetriz ou enfermeira obstétrica.

Métodos não farmacológicos e práticas integrativas e complementares em saúde (PICS) são amplamente adotados. Igualmente, o modelo assistencial garante a presença contínua de até dois acompanhantes, todos os bebês são colocados em contato pele a pele e estimulados à amamentação na primeira hora de vida, a não ser que cuidado emergencial seja necessário. Embora disponíveis, dados sobre estas práticas não foram incluídos neste estudo, pois foram reportados em estudos anteriores realizados no mesmo local^(18-19)^.

A Casa Angela possui ambulância própria para uso imediato em casos de transferências intraparto, pós-parto ou neonatais para o hospital de referência (Hospital Municipal do Campo Limpo Dr. Fernando Mauro Pires da Rocha), localizado a 3 quilômetros, com trajeto aproximado de 10 minutos.

### Período

O período do estudo foi de 2012 a 2021 e incluiu todos os atendimentos de internação de gestantes, partos e transferências maternas e neonatais.

### População e critérios de seleção

Foram incluídas todas as gestantes admitidas para o parto no CPNp no período estudado (n=3.431). Foram excluídas as gestantes cujos prontuários não foram localizados (n=5) e as que pariram durante o trajeto para a Casa, chegando ao serviço para atendimento pós-parto (n=29). A população final foi de 3.397 mulheres.

### Variáveis

A variável de exposição foi a paridade (primíparas, gestantes que, quando admitidas para parto, não haviam parido nenhuma vez, ou multíparas, que já haviam dado à luz a um ou mais bebês anteriormente).

As variáveis de desfecho foram: uso de ocitocina durante o trabalho de parto ou parto (sim ou não); realização de amniotomia (sim ou não); tipo de parto (vaginal não instrumental, fórcipe, cesariana); condição perineal (íntegro ou laceração perineal de 1.º grau, 2.º grau, 3.º grau, 4.º grau ou episiotomia); hemorragia pós-parto (HPP) (sim ou não); transferência materna para o hospital (sim ou não, intraparto ou pós-parto); transferência neonatal (sim ou não); internação neonatal em unidade de cuidados intensivos (UTINeo) (sim ou não) ou intermediários (UCINeo) (sim ou não).

As demais variáveis foram: idade (anos); cor da pele (branca, parda, negra ou amarela); escolaridade (1.º grau incompleto, 1.º grau completo, 2.º grau completo ou superior completo); situação conjugal (vive ou não vive com o/a parceiro/a); renda mensal (≤1, >1≤3, >3≤6 ou >6); tipo de financiamento (negócio social ou SUS); posição no parto (semissentada, quatro apoios, sentada, cócoras, lateral, em pé ou outra); local do parto (cama, banheira, chão, banqueta, chuveiro ou outro); Apgar no 1.º e 5.º minutos (0 a 10); morbimortalidade neonatal relacionada ao parto (descrição da indicação da internação em UTINeo e UCINeo).

### Fontes de informação e instrumento utilizado

As fontes de dados foram os prontuários e os livros de registro de internação, parto e transferência do CPNp, que contêm dados de todos os atendimentos realizados no período do estudo. Para coletar os dados, as autoras elaboraram um instrumento físico com variáveis demográficas e de atendimento clínico, obstétrico e neonatal, que foi preenchido para cada gestante incluída.

### Coleta de dados

A coleta foi realizada por três obstetrizes e uma enfermeira obstétrica treinadas, entre junho 2018 e março 2020. Diante da pandemia COVID-19, a coleta foi interrompida entre 2020 e 2021, retomada em fevereiro de 2022 e concluída em novembro de 2022.

### Tratamento e análise dos dados

Os dados coletados nos instrumentos foram inseridos em planilha do aplicativo *Microsoft Excel* e, posteriormente, transferidos para o programa de análise estatística R v4.3.2 para serem analisados.

As variáveis categóricas foram descritas utilizando-se frequência absoluta e relativa e as variáveis contínuas, com média e desvio-padrão.

Para análise da associação entre a exposição e as variáveis categóricas de desfechos, foi utilizado o teste qui-quadrado e, quando necessário, o qui-quadrado com simulação de Monte Carlo. Para cálculo do *odds ratio* (OR), foram utilizadas as regressões logísticas binária e multinomial. O nível de confiança foi de 95%.

### Aspectos éticos

A pesquisa foi aprovada pelo Comitê de Ética em Pesquisa da Escola de Enfermagem da Universidade de São Paulo (CEP-EEUSP) - parecer n.º 2.026.648 de 2017 - e autorizada pela Comissão Científica da Casa Angela, obtendo dispensa do termo de consentimento livre e esclarecido, pois os dados foram coletados dos prontuários, livros de internação, parto e transferência da instituição.

## Resultados

As 3.397 gestantes que foram atendidas na Casa Angela de 2012 a 2021 eram primíparas em sua maioria (72,2%), tinham em média 27,6 (d.p.=5,4) anos de idade, de cor de pele branca (56,9%), com ensino superior completo (47,2%), em união estável (65,9%) e com renda média mensal entre 1 e 3 salários-mínimos (49,8%). Os partos ocorreram, principalmente, na posição semissentada (30,9%), na cama (38,2%) e na banheira (24,7%). O n < 3.397 participantes para as variáveis cor da pele, escolaridade, situação conjugal e renda familiar deve-se à ausência da informação nas fontes de dados do estudo, com perda variando de 17,5% (escolaridade) a 23,5% (renda familiar) (Tabela 1).

Dado que as características das mulheres apresentadas na Tabela 1 não foram consideradas exposição, a análise destas variáveis não foi estratificada por paridade.

Quanto ao recém-nascido (RN), a média do Apgar no 1.º minuto foi de 8,9 (d.p.=1,2; mínimo=0; máximo=10) e no 5º minuto, 9,7 (d.p.=0,8; mínimo=0; máximo=10), indicando boa vitalidade ao nascer; 95,3% (n=3.236) e 99,4% (n=3.375) apresentaram Apgar ≥ 7 no 1.º e 5.º minutos, respectivamente (dados não mostrados em tabela).

**Tabela 1 -:** Características das mulheres admitidas na Casa Angela. São Paulo, SP, Brasil, 2012-2021

**Característica**	**Média (d.p.) – Mínimo-Máximo**
Idade (anos) (N=3.397)	27,6 (5,4) – 14-46
	N (%)
Paridade	3.397 (100)
Primípara	2.451 (72,2)
Multípara	946 (27,8)
Cor da pele	2.796 (100)
Branca	1.591 (56,9)
Parda	785 (28,1)
Preta	310 (11,1)
Amarela	85 (3,0)
Indígena	25 (0,9)
Escolaridade	2.854 (100)
Ensino superior completo	1.346 (47,2)
Ensino médio completo	1.285 (45,0)
Ensino fundamental completo	162 (5,7)
Ensino fundamental incompleto	61 (2,1)
Situação conjugal	2.830 (100)
Com união estável	1.865 (65,9)
Sem união estável	965 (34,1)
Renda familiar (salários-mínimos)*	2.610 (100)
≤ 1	268 (10,3)
1 ┤ 3	1.302 (49,8)
3 ┤ 6	688 (26,4)
> 6	352 (13,5)
Posição no parto ^†^	2.746 (100)
Semissentada	851 (30,9)
Quatro apoios	488 (17,8)
Sentada	469 (17,1)
Cócoras	437 (15,9)
Lateral	298 (10,9)
Em pé	110 (4,0)
Outra ^‡^	93 (3,4)
Local do parto ^†‡^	2.746 (100)
Cama	1.048 (38,2)
Banheira	679 (24,7)
Chão	448 (16,3)
Banqueta	428 (15,6)
Chuveiro	107 (3,9)
Outro ^§^	8 (0,3)
Fora do quarto PPP ^||^	28 (1,0)

*Corresponde ao salário-mínimo do ano em que a mulher foi atendida na Casa Angela, de 2012 a 2021, com variação de R$622,00 a R$1.100,00, no período; ^†^Inclui apenas as mulheres que deram à luz na Casa Angela; ^‡^Supina, joelhos, “pastor” (um joelho elevado e o outro apoiado); ^§^Cadeira, poltrona, cavalinho; ^||^Pré-parto, parto e pós-parto

Os resultados da assistência e os desfechos maternos analisados segundo a paridade são apresentados na Tabela 2. A taxa total de transferência materna (intraparto e pós-parto) foi de 21,8% (n=738).

**Tabela 2 -:** Assistência e desfechos maternos segundo a paridade das mulheres admitidas na Casa Angela. São Paulo, SP, Brasil, 2012-2021

**Variável**	**Primípara**	**Multípara**	**Total**	**valor-p**
	**N (%)**	**N (%)**	**N (%)**	
Ocitocina	2.451 (100)	946 (100)	3.397 (100)	<0,001*
Sim	156 (6,4)	18 (1,9)	174 (5,1)	
Não	2.295 (93,6)	928 (98,1)	3.223 (94,9)	
Amniotomia	2.451 (100)	946 (100)	3.397 (100)	<0,001*
Sim	339 (13,8)	86 (9,1)	425 (12,5)	
Não	2.112 (86,2)	860 (90,9)	2.972 (87,5)	
Tipo de parto	2.451 (100)	946 (100)	3.397 (100)	<0,001*
Vaginal não instrumental	2.147 (87,6)	935 (98,8)	3.082 (90,8)	
Cesariana	270 (11,0)	10 (1,1)	280 (8,2)	
Fórcipe	34 (1,4)	1 (0,1)	35 (1,0)	
Condição perineal ^†^	2.085 (100)	915 (100)	3.000 (100)	<0,001 ^‡^
Períneo íntegro	226 (10,8)	242 (26,4)	468 (15,6)	
Laceração 1.º grau ^§^	1.177 (56,6)	545 (59,6)	1.722 (57,4)	
Laceração 2.º grau	591 (28,3)	125 (13,7)	716 (23,9)	
Laceração 3.º grau	13 (0,6)	1 (0,1)	14 (0,5)	
Laceração 4.º grau ^||^	-	1 (0,1)	1 (0,0)	
Episiotomia ^||^	78 (3,7)	1 (0,1)	79 (2,6)	
Hemorragia pós-parto ^¶^	1.853 (100)	893 (100)	2.746 (100)	0,023*
Sim	184 (9,9)	65 (7,3)	249 (9,1)	
Não	1.669 (90,1)	828 (92,7)	2.497 (90,9)	
Transferência	2.451 (100)	946 (100)	3.397 (100)	
Intraparto	598 (24,4)	53 (5,6)	651 (19,2)	<0,001*
Pós-parto	66 (2,7)	21 (2,2)	87 (2,6)	0,092*

*Teste qui-quadrado; ^†^Inclui dados parciais dos partos hospitalares; ^‡^Simulação de Monte Carlo; ^§^Inclui lacerações vulvares; ^||^Partos no hospital; ^¶^Inclui dados apenas das mulheres que deram à luz na Casa Angela

Em relação às mulheres que tiveram HPP, 58 (23,3%) foram transferidas para o hospital por alterações nos sinais vitais ou anemia grave (dosagem de hemoglobina abaixo de 7,0 g/dL). Todas as mulheres que tiveram laceração cervical (n=6; 0,2%) e 8 das 14 mulheres que tiveram laceração perineal de 3.º grau na Casa Angela foram transferidas para o hospital no pós-parto para receberem cuidado médico especializado (dados não mostrados em tabela).

A taxa de transferência neonatal foi de 3,3% (n=113), sendo 3,3% (n=81) de RN de primíparas e 3,4% (n=32) de RN de multíparas (p=0,910, teste qui-quadrado) (dados não mostrados em tabela).

Dentre os RN transferidos para o hospital, 88 (77,9%) foram internados no AC para fototerapia, avaliação médica ou realização de exames, tendo alta do hospital ou retornando à Casa Angela após o atendimento (dados não mostrados em tabela).

Do total de 3.397 mulheres, 84 (2,5% ou 25/1.000) tiveram o RN internado na UTINeo ou na UCINeo, com diferença estatisticamente significante em relação à paridade, conforme mostra a Tabela 3. Na mesma tabela estão descritas as indicações para a internação na UTINeo ou UCINeo. As indicações para realização de exames complementares e rotina hospitalar agrupam RN com suspeita de malformação ou patologia a ser esclarecida; em geral, são procedimentos que seguem o protocolo específico do serviço de neonatologia indicado para cada caso.

Além das morbidades descritas na Tabela 3, também ocorreram 11 (0,40%) casos de fratura de clavícula e 2 (0,07%) de lesão de plexo braquial. Houve 2 (0,07%) natimortos e 2 (0,06%) óbitos neonatais precoces.

**Tabela 3-:** Internação do recém-nascido em UTINeo* e UCINeo^†^e indicações (N = 3.397). São Paulo, SP, Brasil, 2012-2021

Variável	Primípara	Multípara	Total	valor-p
	**N=78 (%)**	**N=6 (%)**	**N=84 (%)**	
Sem internação na UTI/UCI	2.373 (69,85) ^‡^	940 (27,67) ^‡^	3.313 (97,5)	<0,001 ^§^
Internação na UTINeo*	53 (1,56) ^‡^	5 (0,15) ^‡^	58 (1,7)	
Internação na UCINeo ^†^	25 (0,74) ^‡^	1 (0,03) ^‡^	26 (0,8)	
Indicação da Internação na UTINeo/UCINeo			N=84 (100%)	
Desconforto respiratório			24 (28,6)	
Infecção ou risco infeccioso/antibioticoterapia			18 (21,4)	
Asfixia/hipoxia perinatal			10 (11,9)	
Apgar baixo			9 (10,7)	
Realização de exames complementares			9 (10,7)	
Síndrome de aspiração de mecônio			8 (9,5)	
Icterícia			4 (4,8)	
Convulsão por encefalopatia			1 (1,2)	
Rotina hospitalar			1 (1,2)	

*Unidade de terapia intensiva neonatal; ^†^Unidade de cuidados intermediários neonatal; ^‡^Devido ao % reduzido, os valores são apresentados com duas casas decimais; ^§^Teste qui-quadrado

Os desfechos maternos que mostraram diferença estatisticamente significante foram analisados por modelos de regressão, com cálculo do OR e IC 95% (Tabela 4).

Entre as primíparas, a chance de receber ocitocina e amniotomia é 3,5 e 1,6 vezes maior que as multíparas, respectivamente. A chance de ter laceração de 2.º grau, ter HPP e de ser transferida intraparto é também maior entre as primíparas (2,6, 40,4 e 5,5 vezes, respectivamente). Para a cesariana, fórcipe e episiotomia, a chance foi igualmente maior entre as primíparas (11,8, 14,8 e 43,7, respectivamente), apesar de IC 95% amplo (Tabela 4).

Quanto à internação do RN em UTINeo ou UCINeo, os resultados mostraram que a chance de ter o bebê internado nessas unidades neonatais foi 5,2 vezes maior entre as primíparas (Tabela 4).

**Tabela 4 -:** Odds ratio (OR) e intervalo de confiança 95% (IC 5%) dos desfechos maternos e internação neonatal segundo a paridade das mulheres admitidas para parto na Casa Angela. São Paulo, SP, Brasil, 2012-2021

**Desfecho (n=3.397)**	**OR (IC 95%)**	**valor-p**
Ocitocina	3,5 (2,2-5,9)	<0,001*
Amniotomia	1,6 (1,3-2,1)	<0,001*
Cesariana	11,8 (6,2-22,2)	<0,001 ^†^
Fórcipe	14,8 (2,0-108,3)	0,008 ^†^
Laceração perineal de 2º grau	2,6 (2,1-3,8) ^‡^	<0,001 ^†^
Episiotomia	43,7 (6,1-314,2) ^‡^	<0,001 ^†^
Hemorragia pós-parto	40,4 (1,1-1,9)	0,024*
Transferência intraparto	5,5 (4,1-7,4)	<0,001 ^†^
Internação do recém-nascido em UTINeo/UCINeo ^§^	5,2 (2,4-13,3)	<0,001*

*Regressão logística binária; ^†^Regressão logística multinominal; ^‡^Calculado contra períneo íntegro e laceração perineal de 1.º grau juntos; ^§^Unidade de terapia intensiva neonatal/Unidade de cuidados intermediários neonatal

## Discussão

Este estudo apresenta os resultados do processo assistencial ao parto no modelo de CPNp com base na caracterização da Casa Angela, da população atendida, do cuidado prestado e dos desfechos maternos e neonatais ao longo de 10 anos de seu funcionamento. Dada a escassez e subutilização dos CPNp no Brasil, estes resultados buscam contribuir para a difusão do modelo, expansão, incentivo e criação de novos CPNp.

Dados disponíveis sobre a caracterização de mulheres atendidas diferem tanto em artigos publicados nacionalmente quanto internacionalmente, o que dificulta a comparação dos resultados. Em relação à cor da pele, diferentemente dos achados deste estudo, em Casas de Parto em Minas Gerais e no Rio de Janeiro, a maior parte das mulheres se declarou preta ou parda^(6,8)^; porém, em estudos no Reino Unido e Estados Unidos, a etnia branca (*white British, white European* ou *any other white ethnicity*) foi declarada por mais de 88% das mulheres^(14,20-21)^.

O perfil das usuárias da Casa Angela em relação à cor de pele, situação conjugal e escolaridade sugere acesso limitado das pessoas em situação de vulnerabilidade, indicando a necessidade de promover políticas e ações locais para ampliar a divulgação do serviço.

Quanto à paridade das mulheres, os dados corroboram com estudo recente realizado no Rio de Janeiro^(6)^ e na Austrália^(22)^, com maior prevalência de primíparas, porém divergem de estudos no Reino Unido^(14,23)^. Os resultados da pesquisa “*Birthplace*”, que analisou desfechos maternos e neonatais de mulheres com gravidez de baixo risco, reportaram que as primíparas eram minoria nos CPNp no Reino Unido e que 36% das primíparas que escolheram dar à luz em Casas de Parto foram transferidas para o hospital durante o trabalho de parto^(15)^. Este achado pode desencorajar primíparas a escolherem esse modelo como primeira opção para dar à luz, pois cerca de 1/3 delas acabam parindo no hospital, dado que diverge deste estudo.

Na Casa Angela, as posições no parto foram diversas, com predomínio das verticalizadas. Isso corrobora a prática assistencial no modelo dos CPN brasileiros, que incentiva e apoia as escolhas da mulher. No período expulsivo, posições supinas (dorsal, ginecológica e litotômica) devem ser evitadas, pois estão associadas a maior ocorrência de anormalidades nos batimentos cardíacos fetais e da prática de episiotomia, além de dificultar o parto vaginal espontâneo^(24)^. Os estudos recentes em CPNp não reportam a posição do parto escolhida pelas mulheres, talvez por indisponibilidade da informação nos prontuários ou pela não associação das posições com desfechos de morbimortalidade, que comumente são o foco dos estudos.

Da mesma forma, os CPN incentivam o parto em locais diferentes da cama, que frequentemente é reportado como o principal local para dar à luz. O segundo local onde as mulheres mais deram à luz na Casa Angela foi na banheira. O parto na água reduz o uso de analgésicos e anestésicos pelo efeito relaxante, o que causa alívio da dor durante a contração. Também diminui a prática da episiotomia por proporcionar o *hands off* (parto que acontece sem que o profissional toque na região perineal da mulher), reduz a chance de HPP, aumenta a chance de períneo íntegro e a satisfação da mulher com o atendimento ao parto. Além disso, ter o parto na água não altera nenhum resultado clínico do RN^(25)^.

Intercorrências como lacerações cervicais ou perineais graves são raras em Casas de Parto. Vale ressaltar que, no período estudado, nenhuma episiotomia foi realizada na Casa Angela, mas taxas entre 1,0% e 8,3% foram reportadas em outros CPNp^(6,22-23)^. A prática de não-episiotomia está embasada em estudos científicos sobre protocolos de não-episiotomia, já que tal intervenção não previne lacerações graves^(26)^.

Vale considerar que práticas menos intervencionistas têm influência sobre traumas perineais. Assim, as taxas de episiotomia são menores e há maior integridade perineal em partos que ocorrem em CPN^(19)^. A classificação do grau de laceração pode divergir, mas mediante treinamento da equipe, pode ser aperfeiçoada^(27)^. Na Casa Angela, escoriações perineais, vulvares e lacerações em lábio interno são classificadas como lacerações de 1.º grau.

Em geral, as taxas de lacerações de 3º e 4º graus são baixas, mas os dados deste estudo diferem dos encontrados em revisão sistemática de países de alta renda, onde a taxa foi de 2,7%^(28)^. A diferença observada precisa ser mais bem explorada, mas pode estar ligada à (in)disponibilidade dos dados perineais das mulheres transferidas intraparto para o hospital ou à subnotificação.

Não foram encontrados estudos que relatam a ocorrência de lacerações cervicais. Na Casa Angela, lacerações cervicais e perineais graves são motivo de transferência pós-parto.

Em relação à HPP, apesar de ter ocorrido em 9,1% dos partos neste estudo, somente 2,1% das mulheres precisaram ser transferidas para o hospital por este motivo. Estudos internacionais reportam dados semelhantes: na Austrália, 3,4% das mulheres que tiveram HPP foram transfundidas no pós-parto^(22)^; a perda sanguínea superior a 1.000 ml gira em torno de 1,2% dos partos em CPNp localizados em países de alta renda^(28)^.

Como as medicações para prevenção e controle da HPP fazem parte dos protocolos assistenciais^(12,16)^, quando não há alteração de sinais vitais ou diagnóstico de anemia pós-hemorragia, os casos podem ser tratados e permanecer aos cuidados dos profissionais da Casa de Parto, sem necessidade de transferência para o hospital.

Estudo sobre fatores de risco para a internação hospitalar por HPP, definida como perda sanguínea > 1000 ml, classificou como médio o risco para HPP de quem tem cicatriz uterina ou mais de três partos vaginais anteriores^(29)^. Não foram encontrados estudos que relacionem a paridade como fator independente para HPP, especialmente em CPNp. No entanto, o presente estudo encontrou que ser primípara aumenta a chance de HPP. Este achado pode estar ligado ao fato de que os fatores de risco anteparto para a HPP tornam a gestante inelegível para parto na Casa de Parto. Deste modo, em CPNp prevalecem os fatores de risco intraparto para a ocorrência de HPP, como trabalho de parto prolongado, trauma perineal de 3.º e 4.º graus e parada de progressão, que são mais comuns em primíparas do que em multíparas^(30)^.

Em relação ao tipo de parto, sabe-se que altas taxas de cesariana não são benéficas e elevam a morbimortalidade materna, especialmente em países de média e baixa renda^(31)^. Segundo a OMS, taxas entre 10 a 15% seriam ideais para que os resultados da cirurgia sejam benéficos e gerem experiência positiva^(32)^. Nesse sentido, a taxa de cesariana de 58% no Brasil^(1)^ deve ser analisada criticamente, pois está muito além da recomendada.

Os CPN foram implementados no SUS para, além de promover a redução das taxas de cesariana, qualificar a assistência obstétrica e proporcionar uma assistência humanizada, que respeita a autonomia da mulher e o processo fisiológico do parto^(5)^. A seleção adequada das gestações de risco habitual, profissionais treinados e capacitados, e o ambiente do CPNp (estrutura que favorece o parto fisiológico, diferente da hospitalar), pode resultar em partos com menos intervenções, sem comprometer os desfechos maternos e neonatais^(33)^.

No presente estudo, a taxa de parto normal foi de 90,8%. Revisão sistemática em países de alta renda reporta taxa de parto normal de 83% dos atendimentos iniciados em CPN em comparação a 61,7% dos iniciados no hospital^(28)^. Estudo australiano reportou que ser atendida em trabalho de parto em CPN aumenta em três vezes a chance de ter um parto normal^(22)^. Da mesma forma, uma revisão integrativa, que analisou 56 estudos, mostrou que os partos que ocorreram em CPNp apresentam um número significativamente menor de intervenções^(7)^. Portanto, a escolha pelo local de parir impacta diretamente no tipo do parto; ou seja, planejar o parto fora do ambiente hospitalar aumenta a chance de parto normal e diminui a cesariana e o parto instrumental^(22,28)^.

As Diretrizes de Assistência ao Parto Normal no Brasil e o *Intrapartum Care Guideline*, do Reino Unido, orientam o uso criterioso da ocitocina e amniotomia^(16-17)^. O uso da ocitocina neste estudo foi ainda menor do que em outros CPNp brasileiros, onde a administração de ocitocina ocorreu em 27,5-30,7% dos casos^(6,8)^. Porém, a prática da amniotomia foi maior do que a de CPNp do Rio de Janeiro (1,2%)^(6)^. Não foi possível comparar estes dados com estudos conduzidos em países de alta renda, pois a ocitocina não está disponível para uso durante trabalho de parto nos CPNp, apenas para prevenção e manejo da HPP. Ao se identificar a necessidade de condução do trabalho de parto, a mulher é transferida para o hospital^(17)^.

A taxa de transferência intraparto varia muito nos estudos nacionais e internacionais (3,6%-37,4%)^(8,15,34-36)^, com maiores taxas em primíparas (22,5%-34,1%) que em multíparas (2,9%-12,2%)^(22,34)^. Essa grande variação precisa ser mais bem explorada, mas pode estar associada às individualidades de cada instituição, como localização, proximidade e acordos com o hospital de referência, profissionais de atenção ao parto, protocolos de assistência e transferência, disponibilidade de recursos humanos e materiais, entre outros.

Em relação às transferências pós-parto e neonatais, observa-se menor discrepância entre os estudos. A transferência pós-parto gira em torno de 1,0% e as neonatais, entre 6,1% e 6,9%^(6,35)^.

Os bons resultados do atendimento às mulheres admitidas para parto na Casa Angela independem da paridade, ou seja, as intervenções e desfechos maternos e neonatais indicam o atendimento às recomendações para o sucesso do modelo^(5,12,16,28)^. Esta conclusão é sustentada por estudos de abrangência nacional e internacional comparativos entre mulheres de risco habitual que planejaram o parto em Casas de Parto versus as que planejaram o parto hospitalar^(8,15,21-22)^.

Em relação ao RN, destacam-se excelentes índices de Apgar, corroborando aqueles encontrados em diferentes Casas de Parto brasileiras, onde 95,2%-99,6% dos bebês receberam Apgar ≥ 7 no 1º e 98,9%-99,9% no 5.º minuto^(6,8)^. Estudo norte-americano recente também encontrou 97,9% dos bebês com Apgar ≥ 7 no 5.º minuto de vida^(20)^. Em CPNp, os fatores associados ao Apgar < 7 no 5º minuto incluem as intercorrências de distocia de ombro, prolapso de cordão, sangramento intraparto e estado fetal não tranquilizador^(8)^ e, em estudos comparativos, os índices de Apgar se assemelham aos encontrados em bebês de baixo risco nascidos no hospital^(6,20)^.

No que diz respeito à transferência neonatal, no CPNp os recursos são restritos ao atendimento de baixo risco; sendo assim, em casos em que há necessidade de avaliação médica, exames complementares ou tratamentos específicos, como a fototerapia, o RN precisa ser transferido para o hospital. Tais motivos não estão diretamente relacionados à assistência ao parto. Como resultado, neste estudo, a grande maioria dos RN (77,9%) foi transferida por motivos não relacionados ao parto e internada em AC, sem apresentar repercussões clínicas graves ou necessidade de internação em UTINeo ou UCINeo.

Em relação às internações em UTINeo ou UCINeo, resultados semelhantes foram encontrados em estudos internacionais (1,2%-2,6%), alguns sem especificação de cuidado intensivo ou intermediário^(28)^. No presente estudo, os principais motivos de internação nessas unidades assistenciais foram desconforto respiratório, infecção ou risco infeccioso, Apgar baixo, asfixia ou hipóxia perinatal, icterícia e síndrome de aspiração de mecônio.

Apesar de observarmos que a chance de o RN ser internado em UTINeo ou UCINeo foi 5,2 vezes maior entre nulíparas, estudo sobre CPN intra e peri-hospitalares no Reino Unido não encontrou diferença em relação à paridade^(37)^.

A morbidade neonatal é uma das principais preocupações na assistência ao parto em CPNp e sinaliza a importância da qualificação das obstetrizes e enfermeiras obstétricas para a avaliação e cuidados imediatos ao RN.

Igualmente acontece com o óbito neonatal. Este estudo encontrou taxa de 0,06%, enquanto a revisão sistemática de países de alta renda encontrou 0,02%^(28)^ e estudo norte-americano 0,03%^(20)^. Porém, estudo em CPNp australiano reportou taxa de 0,3%^(36)^, cinco vezes maior que o presente estudo.

Vale ressaltar que, apesar das diferenças encontradas nas taxas de morbimortalidade entre os estudos em CPNp, estas não são diferentes das encontradas em bebês de mulheres de risco habitual que têm parto em ambiente hospitalar^(13)^.

Apenas dois casos de natimorto (0,06%) ocorreram na Casa Angela durante o período analisado por este estudo, três vezes maior que o dado da revisão sistemática de estudos de países de alta renda, que reportou 0,02% de natimortalidade^(28)^. No entanto, estudo australiano reportou porcentagem quatro vezes maior, 0,24%^(22)^.

Os dados de mortalidade devem ser analisados com cautela devido à raridade do acontecimento e ao tamanho amostral dos estudos. A taxa de partos ocorridos em CPNp gira em torno de 0,3% no Brasil^(1)^, o que inviabiliza um cálculo amostral tendo a mortalidade neonatal como desfecho primário; assim, quando uma morte acontece em estudo com amostra não calculada para este desfecho, o dado reportado pode estar enviesado.

A maioria dos estudos se atém aos resultados dos nascimentos no CPNp, sem o seguimento das mulheres e bebês após as transferências para o hospital. Isto prejudica a qualidade dos dados e pode estar relacionado às tensões e desafios, ainda existentes, na relação entre as Casas de Parto e os hospitais de referência.

A paridade, como preditora das intervenções (ocitocina e amniotomia) e desfechos indesejados (cesariana, fórcipe, laceração de 2º grau, episiotomia, HPP, transferência intraparto e internação do RN em UTINeo e UCINeo), aponta que a primiparidade aumenta a chance de sua ocorrência, com exceção da transferência pós-parto. Mesmo as primíparas tendo piores desfechos que as multíparas neste CPNp, estes não são piores do que os desfechos que teriam se optassem por parirem no hospital^(6,8,21-22)^. A relevância deste achado alerta para o cuidado atento às primíparas em relação às multíparas, mas também reforça a qualidade e segurança do atendimento prestado.

Condições importantes para que a assistência ao parto ocorra com menos intervenções e uso adequado de tecnologias são o modelo de atendimento, cultura local, recursos físicos e materiais, organização do trabalho, recursos humanos, liderança e facilidade de mudança e adaptação^(38-39)^. Apesar de que, desde o lançamento das Diretrizes de Assistência ao Parto Normal, recomendando que profissionais de saúde informem que os CPNp são opção segura para mulheres de baixo risco, o potencial desses estabelecimentos ainda não é utilizado, seja pela subutilização das Casas de Parto existentes, pelo quase inexistente incentivo à criação de novos CPNp, ou pelo não reconhecimento da autonomia das obstetrizes e enfermeiras obstétricas na assistência ao parto. Vale ressaltar que outros países também encontram as mesmas barreiras e dificuldades para a expansão deste modelo de atendimento^(40-41)^.

Este estudo está alinhado com as prioridades em pesquisa para a enfermagem propostas pela Associação Brasileira de Enfermagem, que recomenda a análise de indicadores de atenção nos CPN^(42)^.

No entanto, diante dos bons resultados encontrados neste estudo, devem ser incentivadas mais pesquisas em CPNp. Este local de parto, juntamente com a liderança do cuidado por obstetrizes e enfermeiras obstetras, é potencializador do combate ao modelo biomédico e intervencionista vigente no país.

Destaca-se que, desde sua concepção e criação, estes locais e os profissionais têm como objetivo oferecer e promover um modelo de cuidado biopsicossocial, fisiológico e respeitoso.

Recomendamos que futuros estudos englobem os desfechos das transferências intraparto, pós-parto e neonatais, ressaltando que o modelo de assistência no CPNp é vinculado ao hospital de referência. Não reportar esses resultados desconsidera a existência desta parceria, que torna possível e viável a existência dos CPNp^(39)^.

Para o avanço científico, recomendamos também o desenvolvimento de estudos prospectivos, mesmo conhecendo as dificuldades e o tempo necessário para seu desenvolvimento devido ao baixo número de atendimentos nestes locais. Entendemos que assim colaboramos para o conhecimento e divulgação dos indicadores e a promoção de CPNp como local de parto seguro para o cuidado de mulheres com gestação de risco habitual.

As limitações do estudo estão relacionadas principalmente ao desenho transversal, com fontes de dados secundárias (prontuários e livros de registro) e potenciais vieses de informação e de confundimento. Alguns dados referentes às variáveis de caracterização das gestantes, além de local e posição para o parto, sofreram grandes modificações ao longo dos anos. Por esse motivo, não foi possível coletar informações sobre cor da pele, escolaridade, situação conjugal, renda familiar, posição no parto e local do parto de toda a população do estudo, somente das que estavam disponíveis nas fontes de dados.

## Conclusão

Em relação às multíparas, as primíparas têm maior chance de transferência intraparto, uso de ocitocina, amniotomia, laceração perineal de 2.º grau, episiotomia, HPP, fórceps, cesariana e internação do RN em UTINeo ou UCINeo.

Porém, mesmo entre as primíparas, a prática assistencial no CPNp mostra o uso restrito de intervenções – ocitocina, amniotomia, episiotomia –, a prevalência de integridade perineal ou trauma perineal leve (lacerações de 1.º grau) e a opção das mulheres por posições verticalizadas e pelo parto fora da cama. Destaca-se que as baixas taxas de cesariana e fórceps e de morbimortalidade materna e neonatal são compatíveis com o perfil das mulheres de risco habitual atendidas no local.

Por sua vez, a retaguarda hospitalar é fundamental para o atendimento das mulheres e RN, dado que as transferências maternas e neonatais, mesmo com taxas baixas, são um desfecho previsto no modelo de CPNp.

Sendo assim, os resultados apontam para a segurança do parto na Casa Angela e a relevância das Casas de Parto como local para o parto no Brasil no combate às elevadas taxas de cesariana e de intervenções desnecessárias no parto.

# Translation: Resultados maternos y neonatales de la atención del parto en un Centro de Parto Normal perihospitalario*

**DOI:** 10.1590/1518-8345.7208.4595

**Authors:** Nathalie Leister, Gisele Almeida Lopes, Caroline de Oliveira Ferreira Iguchi, Thalita Vital Botelho, Maria Luiza Gonzalez Riesco

**Affiliations:** 1 Universidade de São Paulo, Escola de Enfermagem, São Paulo, SP, Brasil.; 2 City St George’s, University of London, School of Health and Medical Sciences, Londres, Reino Unido.; 3 Associação Comunitária Monte Azul, Casa Angela - Centro de Parto humanizado, São Paulo, SP, Brasil.

**Keywords:** Centros de Asistencia al Embarazo y al Parto, Entorno del Parto, Partería, Evaluación de Resultado en la Atención de Salud, Estudios Transversales, Parto

## Abstract

comparar los desenlaces de la atención materna y neonatal con la paridad de las mujeres y describir la morbilidad y mortalidad neonatal de los bebés de mujeres que ingresaron en trabajo de parto.

estudio transversal con 3.397 mujeres ingresadas para parto en un Centro de Parto Normal perihospitalario y sus recién nacidos. La variable de exposición fue paridad y los desenlaces fueron uso de oxitocina y amniotomía, tipo de parto, condición perineal, hemorragia postparto, derivaciones maternas y neonatales y hospitalización del recién nacido en unidades de terapia intensiva o cuidados intermedios. Se realizó análisis descriptivo de los datos y regresión logística.

la primiparidad se asoció con mayor probabilidad de recibir oxitocina y amniotomía, ser derivada durante el parto, tener laceración de 2.º grado, episiotomía, hemorragia postparto, cesárea y parto con fórceps e internación del recién nacido en unidades de terapia intensiva o cuidados intermedios. Los partos se produjeron principalmente en posiciones semisentada y erguida, en la cama y en la bañera. La tasa de derivación materna fue del 21,8% y la tasa de derivación neonatal fue del 3,3%.

la primiparidad es un factor predictivo de las intervenciones analizadas y de los desenlaces maternos y neonatales indeseables. Sin embargo, el Centro de Parto Normal perihospitalario estudiado puede considerarse seguro para el parto de mujeres de bajo riesgo.

## Destacados

**(1)** El CPNp es seguro para la atención de mujeres de bajo riesgo y sus bebés. **(2)** Las mujeres primíparas tienen mayor probabilidad de recibir oxitocina y amniotomía que las mujeres multíparas. **(3)** Las mujeres primíparas tienen mayor probabilidad de ser derivadas durante el parto que las mujeres multíparas. **(4)** Las mujeres primíparas tienen mayor probabilidad de sufrir traumatismo perineal que las mujeres multíparas. **(5)** Las mujeres primíparas tienen mayor probabilidad de tener un parto con fórceps y por cesárea que las mujeres multíparas.

## Introducción

En Brasil, el modelo de atención del parto y nacimiento es hospitalocéntrico, intervencionista y medicocéntrico, se caracteriza por tener altas tasas de cesárea, que en 2022 representaron el 58% de los nacimientos^(1)^. En los centros obstétricos, incluso en embarazos de bajo riesgo, la atención generalmente consiste en intervenciones de rutina, no justificadas por razones clínicas u obstétricas, contrarias a las recomendaciones científicas y a las de la Organización Mundial de la Salud (OMS)^(2-3)^.

Este modelo se cuestiona en el país desde la década de 1990, debido al exceso de cesáreas y al estancamiento de las tasas de mortalidad materna y perinatal^(4)^. Desde entonces, se han formulado políticas públicas para mejorar la atención obstétrica en el Sistema Único de Salud (SUS). Entre estas políticas, se destacan la propuesta de crear Centros de Parto Normal (CPN), en 1999, y la política de la Red Cegonha, en 2011 (recientemente reestructurada como Red Alyne), que incluyó un conjunto de cambios para promover una atención de calidad, que cuenta con lugares más adecuados para dar a luz, según la elección de la gestante, y con obstetrices y enfermeras obstétricas responsables de la atención^(5)^.

En estudios descriptivos y observacionales realizados en CPN perihospitalarios (CPNp) brasileños, también llamados Casas de Parto, los resultados apuntan al uso de buenas prácticas, como el cumplimiento del derecho a tener un acompañante durante toda la hospitalización, admisión en la fase activa del parto, uso de métodos no farmacológicos para aliviar el dolor, libre adopción de la posición de parto y uso prudente de prácticas como amniotomía, administración de oxitocina y episiotomía^(6-8)^.

Además, el CPNp promueve la autonomía y la satisfacción de quienes reciben atención^(7,9)^. En virtud de los buenos desenlaces, incluso cuando hay derivaciones maternas o neonatales a centros obstétricos, la demanda del parto en entornos extrahospitalarios está creciendo a nivel internacional^(10)^.

Los buenos resultados de la atención que brindan los CPNp dependen de criterios de elegibilidad y triage adecuados de las mujeres embarazadas de bajo riesgo^(11-12)^. Los estudios revelan que hay resultados favorables independientemente de la paridad, aunque las primíparas tienen mayores tasas de derivación y cesárea, hemorragia posparto o necesidad de transfusión e infección amniótica sospechada o confirmada. A su vez, los bebés tienen mayor probabilidad de presentar Apgar < 7 a los 5 minutos, ingresar en la unidad de terapia intensiva neonatal, sepsis y muerte neonatal^(11,13-15)^.

El Ministerio de Salud de Brasil reconoce los buenos desenlaces maternos y neonatales, por ello lanzó, en 2016, la Guía Nacional de Asistencia al Parto Normal, que recomienda que las gestantes reciban información basada en evidencia científica y en formato accesible sobre los riesgos y beneficios asociados a los lugares de parto^(16)^. A partir de entonces, se esperaba que se incentivara aún más el modelo con la creación de nuevos CPNp y el aumento del número de nacimientos en estos lugares, dado que hay fuerte evidencia de que estos servicios son seguros, tienen mejores resultados maternos y perinatales, reducen las tasas de cesárea, mejoran la satisfacción con el parto y son económicamente más sostenibles que la atención de embarazos y partos de bajo riesgo en centros obstétricos^(6,8-9)^.

Cabe destacar que la Guía Nacional mencionada fue desarrollada en su mayoría en base a estudios y directrices internacionales junto con la literatura brasileña^(2,17)^. En este caso, hay pocos estudios sobre configuración, funcionamiento, atención y desenlaces en los CPNp en Brasil.

Con el fin de contribuir y avanzar en el conocimiento del modelo de atención y difundir los desenlaces maternos y neonatales de la atención que brindan, los objetivos de este estudio son: 1) comparar los resultados de la atención materna y neonatal con la paridad de las mujeres que ingresaron en trabajo de parto; 2) describir datos de morbilidad y mortalidad neonatal en un CPNp brasileño.

## Método

### Tipo de estudio

Estudio observacional transversal analítico, cuyo informe sigue las directrices *Strengthening the Reporting of Observational Studies in Epidemiology* (STROBE) para estudios transversales.

### Lugar

El estudio se realizó en el CPNp *Casa Angela*, *Centro de Parto Humanizado*, ubicado en la zona sur de la ciudad de São Paulo, SP, Brasil. Se puede obtener información detallada sobre el lugar de estudio en el sitio *web* de la institución (https://www.casaangela.org.br/a-casa-angela.html).

*Casa Angela* es administrada por la organización no gubernamental *Associação Comunitária Monte Azul*. Fue fundada en diciembre de 2009 y funcionó parcialmente hasta 2011, brindando atención prenatal, cuidado infantil y talleres para adolescentes. En 2012, el servicio se implementó de manera ininterrumpida, con un modelo de subsidio cruzado y empresas sociales, en el que se financiaba a las mujeres habitantes de la región que no podían pagar la atención mediante el monto cobrado a quienes sí podían pagar el servicio, y también con donaciones y financiamiento de proyectos que desarrollaba la institución. Este modelo funcionó hasta fines de 2015, cuando se estableció una alianza con la Secretaría Municipal de Salud de la ciudad de São Paulo para financiar la atención. A partir de 2020, la atención pasó a ser financiada completamente por el SUS y las usuarias no pagan por el servicio (Figura 1).

**Figura 1 -:** Distribución de la cantidad de servicios prestados por año y tipo de financiamiento (N = 3.397). São Paulo, SP, Brasil, 2012-2021

Las personas atendidas en *Casa Angela* son derivadas por la unidad básica de salud (UBS) o llegan por demanda espontánea. Las mujeres embarazadas se someten a una evaluación de riesgo y, si cumplen con los criterios de elegibilidad, comienzan a recibir atención prenatal en la institución a partir de las 28 semanas de embarazo. El servicio es exclusivo para personas sanas que cumplan los criterios establecidos en el Manual Técnico de las Casas de Parto del Municipio de São Paulo, documento adoptado como protocolo institucional^(12)^. El equipo clínico está conformado por 20 obstetrices y enfermeras obstétricas y nueve técnicas en enfermería.

La admisión para el parto se realiza después de una evaluación de riesgo y una reevaluación de elegibilidad. La atención del parto se brinda en la sala PPP (preparto, parto y postparto), donde la parturienta/puérpera, el bebé y sus acompañantes permanecen desde el parto hasta 2 horas después del parto. Después del parto, la familia es enviada a una habitación privada en un alojamiento compartido (AC) y el alta se produce alrededor de 24 horas después del parto. Se programan dos consultas posparto con la obstetriz o enfermera obstétrica.

Los métodos no farmacológicos y las prácticas integradoras y complementarias de salud (PICS) se adoptan ampliamente. Asimismo, el modelo de atención garantiza la presencia continua de hasta dos acompañantes, todos los bebés se ponen en contacto piel con piel y se los estimula para que mamen en la primera hora de vida, salvo que requieran atención de emergencia. Aunque estaban disponibles, los datos sobre estas prácticas no se incluyeron en este estudio porque se informaron en estudios previos realizados en el mismo lugar^(18-19)^.

*Casa Angela* cuenta con ambulancia propia para uso inmediato en casos de derivaciones durante el parto, posparto o neonatales al hospital de referencia (*Hospital Municipal do Campo Limpo Dr. Fernando Mauro Pires da Rocha*), ubicado a 3 kilómetros, con un tiempo aproximado de trayecto de 10 minutos.

### Período

El periodo de estudio fue de 2012 a 2021 e incluyó todas las hospitalizaciones de gestantes, los partos y las derivaciones maternas y neonatales.

### Población y criterios de selección

Se incluyeron todas las gestantes ingresadas para parto en el CPNp durante el periodo estudiado (n=3.431). Se excluyeron las mujeres embarazadas cuyos registros médicos no fueron localizados (n=5) y las que dieron a luz durante el trayecto a la institución y llegaron al servicio para recibir atención postparto (n=29). La población final estuvo compuesta por 3.397 mujeres.

### Variables

La variable de exposición fue la paridad (primíparas, embarazadas que al momento del ingreso para el parto nunca habían dado a luz, o multíparas, que ya habían dado a luz uno o más bebés previamente).

Las variables de desenlace fueron: uso de oxitocina durante el trabajo de parto o el parto (sí o no); realización de amniotomía (sí o no); tipo de parto (vaginal no instrumental, fórceps, cesárea); condición perineal (intacto o laceración perineal de 1^er^ grado, 2.º grado, 3^er^ grado, 4.º grado o episiotomía); hemorragia posparto (HPP) (sí o no); traslado materno al hospital (sí o no, intraparto o postparto); derivación neonatal (sí o no); ingreso neonatal a la unidad de terapia intensiva (UTIN) (sí o no) o unidad de cuidados intermedios (UCIN) (sí o no).

Las otras variables fueron: edad (años); color de piel (blanca, morena, negra o amarilla); educación (primaria incompleta, primaria completa, secundaria completa o superior completa); estado civil (vive o no con la pareja); ingreso mensual (≤1, >1≤3, >3≤6 o >6); tipo de financiación (empresas sociales o SUS); posición durante el parto (semisentada, cuadrupedia, sentada, en cuclillas, de lado, de pie u otra); lugar del parto (cama, bañera, suelo, banco, ducha u otro); Apgar al 1^er^ y 5.º minuto (0 a 10); morbilidad y mortalidad neonatal relacionada con el parto (descripción de la indicación del ingreso a la UTIN y UCIN).

### Fuentes de información e instrumento utilizado

Las fuentes de datos fueron las historias clínicas y los registros de internación, parto y derivación del CPNp, que contienen datos de toda la atención brindada durante el periodo de estudio. Para la recolección de datos, las autoras desarrollaron un instrumento físico con variables demográficas y de atención clínica, obstétrica y neonatal, que fue completado para cada una de las gestantes incluidas.

### Recolección de datos

La recolección fue realizada por tres obstetrices y una enfermera obstétrica capacitadas, entre junio de 2018 y marzo de 2020. Debido a la pandemia de COVID-19, la recolección se interrumpió entre 2020 y 2021, se reanudó en febrero de 2022 y concluyó en noviembre de 2022.

### Procesamiento y análisis de datos

Los datos recolectados de los instrumentos fueron ingresados en una hoja de cálculo de *Microsoft Excel* y posteriormente transferidos al programa de análisis estadístico R v4.3.2 para ser analizados.

Las variables categóricas se describieron mediante frecuencia absoluta y relativa y las variables continuas, con media y desviación estándar.

Para analizar la asociación entre la exposición y las variables categóricas de desenlace, se utilizó la prueba de chi-cuadrado y, cuando fue necesario, la prueba de chi-cuadrado con simulación de Monte Carlo. Para calcular el *odds ratio* (OR), se utilizaron regresiones logísticas binarias y multinomiales. El intervalo de confianza fue del 95% (IC 95%).

### Aspectos éticos

La investigación fue aprobada por el Comité de Ética en Investigación de la Escuela de Enfermería de la Universidad de São Paulo (CEP-EEUSP) con dictamen n.º 2.026.648 de 2017 y autorizada por el Comité Científico de *Casa Angela*. No fue necesario completar el formulario de consentimiento libre e informado, dado que los datos fueron recolectados de las historias clínicas, los registros de internación, parto y derivación de la institución.

## Resultados

Las 3.397 mujeres embarazadas atendidas en *Casa Angela* de 2012 a 2021 fueron en su mayoría primíparas (72,2%), tenían en promedio 27,6 (DE=5,4) años de edad, color de piel blanca (56,9%), tenían educación superior completa (47,2%), estaban en unión estable (65,9%) y tenían un ingreso mensual promedio entre 1 y 3 salarios mínimos (49,8%). Los partos ocurrieron principalmente en posición semisentada (30,9%), en la cama (38,2%) y en la bañera (24,7%). El hecho de que el número de participantes sea n < 3.397 para las variables color de piel, educación, estado civil e ingreso familiar se debe a la falta de información en las fuentes de datos del estudio, con pérdidas que van desde 17,5% (educación) hasta 23,5% (ingreso familiar) (Tabla 1).

Dado que las características de las mujeres presentadas en la Tabla 1 no fueron consideradas exposición, el análisis de estas variables no fue estratificado por paridad.

Con respecto al recién nacido (RN), el puntaje de Apgar promedio en el 1^er^ minuto fue de 8,9 (DE=1,2; mínimo=0; máximo=10) y en el 5.º minuto, de 9,7 (DE=0,8; mínimo=0; máximo=10), lo que indica buena vitalidad al nacer; el 95,3% (n=3.236) y el 99,4% (n=3.375) tuvieron Apgar ≥ 7 en el 1^er^ y 5.º minuto, respectivamente (datos no incluidos en la tabla).

**Tabla 1-:** Características de las mujeres ingresadas en *Casa Angela*. São Paulo, SP, Brasil, 2012-2021

**Característica**	**Media (DE) – Mínimo-Máximo**
Edad (años) (N=3.397)	27,6 (5,4) – 14-46
	N (%)
Paridad	3.397 (100)
Primípara	2.451 (72,2)
Multípara	946 (27,8)
Color de piel	2.796 (100)
Blanco	1.591 (56,9)
Moreno	785 (28,1)
Negro	310 (11,1)
Amarillo	85 (3,0)
Indígena	25 (0,9)
Educación	2.854 (100)
Educación superior completa	1.346 (47,2)
Educación secundaria completa	1.285 (45,0)
Educación primaria completa	162 (5,7)
Educación primaria incompleta	61 (2,1)
Estado civil	2.830 (100)
En unión estable	1.865 (65,9)
Sin unión estable	965 (34,1)
Ingreso familiar (salarios mínimos)*	2.610 (100)
≤ 1	268 (10,3)
1 ┤ 3	1.302 (49,8)
3 ┤ 6	688 (26,4)
> 6	352 (13,5)
Posición de parto ^†^	2.746 (100)
Semisentada	851 (30,9)
Cuadrupedia	488 (17,8)
Sentada	469 (17,1)
Cuclillas	437 (15,9)
De lado	298 (10,9)
De pie	110 (4,0)
Otra ^‡^	93 (3,4)
Lugar del parto ^†‡^	2.746 (100)
Cama	1.048 (38,2)
Bañera	679 (24,7)
Piso	448 (16,3)
Banco	428 (15,6)
Ducha	107 (3,9)
Otro ^§^	8 (0,3)
Fuera de la sala PPP ^||^	28 (1,0)

*Corresponde al salario mínimo del año en que la mujer fue atendida en *Casa Angela*, de 2012 a 2021, y oscila entre R$ 622,00 y R$ 1.100,00 en ese período; ^†^Incluye sólo mujeres que dieron a luz en *Casa Angela*; ^‡^Supina, rodillas, “pastor” (una rodilla levantada y la otra apoyada); ^§^Silla, sillón; ^||^Preparto, parto y posparto

Los resultados de la atención y los desenlaces maternos analizados según la paridad se presentan en la Tabla 2. La tasa de derivación materna total (intraparto y posparto) fue del 21,8% (n=738).

**Tabla 2 -:** Atención materna y desenlaces según la paridad de las mujeres ingresadas en *Casa Angela*. São Paulo, SP, Brasil, 2012-2021

**Variable**	**Primípara**	**Multípara**	**Total**	**valor p**
	**N (%)**	**N (%)**	**N (%)**	
Oxitocina	2.451 (100)	946 (100)	3.397 (100)	<0,001*
Sí	156 (6,4)	18 (1,9)	174 (5,1)	
No	2.295 (93,6)	928 (98,1)	3.223 (94,9)	
Amniotomía	2.451 (100)	946 (100)	3.397 (100)	<0,001*
Sí	339 (13,8)	86 (9,1)	425 (12,5)	
No	2.112 (86,2)	860 (90,9)	2.972 (87,5)	
Tipo de parto	2.451 (100)	946 (100)	3.397 (100)	<0,001*
Vaginal no instrumental	2.147 (87,6)	935 (98,8)	3.082 (90,8)	
Cesárea	270 (11,0)	10 (1,1)	280 (8,2)	
Fórceps	34 (1,4)	1 (0,1)	35 (1,0)	
Estado perineal ^†^	2.085 (100)	915 (100)	3.000 (100)	<0,001 ^‡^
Perineo intacto	226 (10,8)	242 (26,4)	468 (15,6)	
Laceración de 1 ^er^ grado ^§^	1.177 (56,6)	545 (59,6)	1.722 (57,4)	
Laceración de 2.º grado	591 (28,3)	125 (13,7)	716 (23,9)	
Laceración de 3 ^er^ grado	13 (0,6)	1 (0,1)	14 (0,5)	
Laceración de 4.º grado ^||^	-	1 (0,1)	1 (0,0)	
Episiotomía ^||^	78 (3,7)	1 (0,1)	79 (2,6)	
Hemorragia posparto ^¶^	1.853 (100)	893 (100)	2.746 (100)	0,023*
Sí	184 (9,9)	65 (7,3)	249 (9,1)	
No	1.669 (90,1)	828 (92,7)	2.497 (90,9)	
Derivación	2.451 (100)	946 (100)	3.397 (100)	
Intraparto	598 (24,4)	53 (5,6)	651 (19,2)	<0,001*
Posparto	66 (2,7)	21 (2,2)	87 (2,6)	0,092*

*Prueba de chi-cuadrado; ^†^Incluye datos parciales de los partos hospitalarios; ^‡^Simulación de Monte Carlo; ^§^Incluye laceraciones vulvares; ^||^Partos en el hospital; ^¶^Incluye datos sólo de las mujeres que dieron a luz en *Casa Angela*

En lo que respecta a las mujeres que presentaron HPP, 58 (23,3%) fueron trasladadas al hospital por presentar alteraciones en los signos vitales o anemia grave (nivel de hemoglobina inferior a 7,0 g/dL). Todas las mujeres que presentaron laceración cervical (n=6; 0,2%) y 8 de las 14 mujeres que presentaron laceración perineal de 3^er^ grado en *Casa Angela* fueron derivadas al hospital después del parto para recibir atención médica especializada (datos no incluidos en la tabla).

La tasa de derivación neonatal fue del 3,3% (n=113), el 3,3% (n=81) fue de RN de mujeres primíparas y el 3,4% (n=32) de RN de mujeres multíparas (p=0,910, prueba de chi-cuadrado) (datos no incluidos en la tabla).

De los RN derivados al hospital, 88 (77,9%) fueron ingresados en el AC para fototerapia, evaluación médica o exámenes, y fueron dados de alta del hospital o volvieron a *Casa Angela* después del tratamiento (datos no incluidos en la tabla).

De las 3.397 mujeres, 84 (2,5% o 25/1.000) tuvieron el RN ingresado en la UTIN o UCIN; se observó diferencia estadísticamente significativa con respecto a la paridad, como se puede ver en la Tabla 3. En la misma tabla se describen las indicaciones de ingreso a la UTIN o UCIN. Las indicaciones para realizar exámenes complementarios y de rutina hospitalaria incluyen RN con sospecha de malformación o patología a determinar; en general, son procedimientos que siguen el protocolo específico del servicio de neonatología, indicado para cada caso.

Además de las morbilidades descritas en la Tabla 3, también hubo 11 (0,40%) casos de fractura de clavícula y 2 (0,07%) de lesión del plexo braquial. Se registraron 2 (0,07%) mortinatos y 2 (0,06%) muertes neonatales precoces.

**Tabla 3 -:** Ingreso de recién nacidos a UTIN* y UCIN^†^e indicaciones (N = 3.397). São Paulo, SP, Brasil, 2012-2021

Variable	Primípara	Multípara	Total	valor p
	**N=78 (%)**	**N=6 (%)**	**N=84 (%)**	
Sin ingreso a UTI/UCI	2.373 (69,85) ^‡^	940 (27,67) ^‡^	3.313 (97,5)	<0,001 ^§^
Ingreso a UTIN*	53 (1,56) ^‡^	5 (0,15) ^‡^	58 (1,7)	
Ingreso a UCIN ^†^	25 (0,74) ^‡^	1 (0,03) ^‡^	26 (0,8)	
Indicación de ingreso a UTIN*/UCIN ^†^			84 (100%)	
Molestias respiratorias			24 (28,6)	
Infección o riesgo de infección/tratamiento con antibióticos			18 (21,4)	
Asfixia/hipoxia perinatal			10 (11,9)	
Apgar bajo			9 (10,7)	
Realización de exámenes complementarios			9 (10,7)	
Síndrome de aspiración de meconio			8 (9,5)	
Ictericia			4 (4,8)	
Convulsión por encefalopatía			1 (1,2)	
Rutina hospitalaria			1 (1,2)	

*Unidad de terapia intensiva neonatal; ^†^Unidad de cuidados intermedios neonatales; ^‡^Debido al % reducido, los valores se presentan con dos decimales; ^§^Prueba de chi-cuadrado

Los desenlaces maternos que mostraron una diferencia estadísticamente significativa se analizaron mediante modelos de regresión, con cálculo de OR e IC del 95% (Tabla 4).

La probabilidad de recibir oxitocina y amniotomía de las mujeres primíparas es 3,5 y 1,6 veces mayor que la de las multíparas, respectivamente. La probabilidad de las mujeres primíparas de tener una laceración de 2.º grado, tener HPP y ser derivada durante el parto también es mayor (2,6, 40,4 y 5,5 veces, respectivamente). En cuanto a la cesárea, el fórceps y la episiotomía, la probabilidad también fue mayor para las mujeres primíparas (11,8, 14,8 y 43,7, respectivamente), a pesar de que un IC del 95% es amplio (Tabla 4).

En lo que respecta a la hospitalización del RN en la UTIN o UCIN, los resultados revelaron que la probabilidad de que el bebé sea ingresado en estas unidades neonatales fue 5,2 veces mayor en el caso de las mujeres primíparas (Tabla 4).

**Tabla 4 -:** Odds ratio (OR) e intervalo de confianza del 95% (IC5%) de los desenlaces maternos y de hospitalización neonatal según la paridad de las mujeres ingresadas para parto en *Casa Angela*. São Paulo, SP, Brasil, 2012-2021

**Desenlace (n=3.397)**	**OR (IC 95%)**	**valor p**
Oxitocina	3,5 (2,2-5,9)	<0,001*
Amniotomía	1,6 (1,3-2,1)	<0,001*
Cesárea	11,8 (6,2-22,2)	<0,001 ^†^
Fórceps	14,8 (2,0-108,3)	0,008 ^†^
Laceración perineal de 2.º grado	2,6 (2,1-3,8) ^‡^	<0,001 ^†^
Episiotomía	43,7 (6,1-314,2) ^‡^	<0,001 ^†^
Hemorragia posparto	40,4 (1,1-1,9)	0,024*
Derivación intraparto	5,5 (4,1-7,4)	<0,001 ^†^
Ingreso del recién nacido a la UTIN/UCIN ^§^	5,2 (2,4-13,3)	<0,001*

*Regresión logística binaria; ^†^Regresión logística multinomial; ^‡^Calculado versus perineo intacto y laceración perineal de 1^er^ grado juntos; ^§^Unidad de terapia intensiva neonatal/Unidad de cuidados intermedios neonatales

## Discusión

Este estudio presenta los resultados del proceso de atención del parto en el modelo de CPNp a partir de la caracterización de *Casa Angela*, la población atendida, la atención brindada y los desenlaces maternos y neonatales a lo largo de 10 años de funcionamiento. Dada la escasez y subutilización del CPNp en Brasil, estos resultados buscan contribuir a la difusión del modelo, expansión, incentivo y creación de nuevos CPNp.

Los datos disponibles sobre la caracterización de las mujeres que recibieron atención difieren tanto en los artículos publicados a nivel nacional como internacional, lo que dificulta comparar los resultados. En cuanto al color de piel, a diferencia de los hallazgos de este estudio, en las Casas de Parto de Minas Gerais y Río de Janeiro, la mayoría de las mujeres declaró que se consideraba negra o morena^(6,8)^; mientras que, en estudios realizados en el Reino Unido y Estados Unidos, el 88% de las mujeres declaró que pertenecía a la etnia blanca^(14,20-21)^ (*white British, white European* o *any other white ethnicity*).

El perfil de los usuarios de *Casa Angela* en relación al color de piel, estado civil y educación sugiere un acceso limitado para personas en situación de vulnerabilidad, lo que indica que es necesario promover políticas y acciones locales para aumentar la difusión del servicio.

Con respecto a la paridad femenina, los datos coinciden con los de un estudio reciente realizado en Río de Janeiro^(6)^ y Australia^(22)^, en los que predominan las mujeres primíparas, pero difieren de los obtenidos en estudios realizados en Reino Unido^(14,23)^. Los resultados del estudio “*Birthplace”*, que analizó los desenlaces maternos y neonatales de mujeres con embarazos de bajo riesgo, informaron que las primíparas eran minoría en los CPNp del Reino Unido y que el 36% de las primíparas que optaron por dar a luz en Casas de Parto fueron derivadas al hospital durante el trabajo de parto^(15)^. Este hallazgo puede disuadir a las madres primerizas de elegir este modelo como primera opción para dar a luz, dado que aproximadamente 1/3 de ellas termina dando a luz en el hospital; dicho dato no coincide con el del presente estudio.

En *Casa Angela* las posiciones de parto adoptadas fueron variadas y predominaron las posiciones verticales. Esto demuestra la práctica asistencial adoptada por el modelo de los CPN brasileños, que incentiva y apoya las elecciones de las mujeres. Durante el período expulsivo hay que evitar las posiciones supinas (dorsal, ginecológica y de litotomía), ya que se asocian a una mayor incidencia de anormalidades en los latidos cardíacos fetales y a la práctica de episiotomía, además de dificultar el parto vaginal espontáneo^(24)^. Estudios recientes sobre CPNp no informan la posición de parto elegida por las mujeres, tal vez debido a la falta de información en las historias clínicas o a que no hay asociación entre las posiciones y los desenlaces de morbilidad y mortalidad, que comúnmente son el foco de los estudios.

De manera similar, los CPN fomentan que el parto no se realice en la cama, que a menudo se considera el principal lugar para dar a luz. El segundo lugar donde más mujeres dieron a luz en *Casa Angela* fue la bañera. El parto en el agua reduce el uso de analgésicos y anestésicos debido a su efecto relajante, que provoca alivio del dolor durante las contracciones. También reduce la práctica de la episiotomía al permitir un parto *hands off* (que ocurre sin que el profesional toque la región perineal de la mujer), reduce las posibilidades de HPP, aumenta las posibilidades de que el perineo siga intacto y la satisfacción de la mujer con la atención del parto. Además, el parto en el agua no altera ningún resultado clínico del RN^(25)^.

Complicaciones como laceraciones cervicales o perineales graves son raras en las Casas de Parto. Cabe destacar que, durante el período estudiado, no se realizaron episiotomías en *Casa Angela*, pero se reportaron tasas entre 1,0% y 8,3% en otros CPNp^(6,22-23)^. La práctica de la no episiotomía se basa en estudios científicos sobre protocolos de no-episiotomía, dado que dicha intervención no previene laceraciones graves^(26)^.

Hay que considerar que las prácticas menos intervencionistas influyen en los traumas perineales. Por ello, las tasas de episiotomía son menores y hay mayor integridad perineal en los partos que ocurren en CPN^(19)^. La clasificación del grado de laceración puede variar, pero con el entrenamiento del equipo se puede mejorar^(27)^. En *Casa Angela* las abrasiones perineales y vulvares y las laceraciones en el labio interno se clasifican como laceraciones de 1^er^ grado.

En general, las tasas de laceraciones de 3^er^ y 4.º grado son bajas, pero los datos de este estudio difieren de los encontrados en una revisión sistemática de países de altos ingresos, donde la tasa fue del 2,7%^(28)^. La diferencia observada debe ser estudiada más a fondo, pero puede estar vinculada a la (in)disponibilidad de los datos perineales de las mujeres derivadas intraparto al hospital o a la subnotificación.

No se encontraron estudios que informen la presencia de laceraciones cervicales. En *Casa Angela*, las laceraciones cervicales y perineales severas son motivo de derivación postparto.

Con respecto a la HPP, aunque se presentó en el 9,1% de los partos en este estudio, sólo el 2,1% de las mujeres debió ser derivada al hospital por este motivo. Estudios internacionales arrojan datos similares: en Australia, el 3,4% de las mujeres que tuvieron HPP recibieron transfusiones posparto^(22)^; una pérdida de sangre superior a 1.000 ml representa alrededor del 1,2% de los partos en CPNp ubicados en países de altos ingresos^(28)^.

Como los medicamentos para prevenir y controlar la HPP son parte de los protocolos de atención^(12,16)^, cuando no hay cambio en los signos vitales o diagnóstico de anemia posthemorrágica, los profesionales de la Casa de Parto pueden tratar y continuar brindándoles atención a esos casos, sin necesidad de derivarlos al hospital.

Un estudio sobre los factores de riesgo de ingreso hospitalario debido a HPP, definida como pérdida de sangre > 1.000 ml, clasificó el riesgo de HPP como medio para las mujeres con una cicatriz uterina o más de tres partos vaginales previos^(29)^. No se encontraron estudios que relacionen la paridad como factor independiente de HPP, especialmente en CPNp. Sin embargo, el presente estudio reveló que ser primípara aumenta el riesgo de HPP. Este hallazgo puede estar relacionado con el hecho de que los factores de riesgo previos al parto de HPP hacen que la mujer embarazada no sea elegible para el parto en la Casa de Parto. Por ende, en los CPNp prevalecen los factores de riesgo intraparto de presencia de HPP, como trabajo de parto prolongado, trauma perineal de 3^er^ y 4.º grado y la detención de la progresión, que son más frecuentes en primíparas que en multíparas^(30)^.

En cuanto al tipo de parto, se sabe que las altas tasas de cesárea no son beneficiosas y aumentan la morbilidad y mortalidad materna, especialmente en países de ingresos medios y bajos^(31)^. Según la OMS, las tasas ideales para que los resultados de la cirugía sean beneficiosos y generen una experiencia positiva serían entre 10 y 15%^(32)^. Por lo tanto, la tasa de cesáreas del 58% en Brasil^(1)^ requiere un análisis crítico, dado que está muy por encima de la tasa recomendada.

Los CPN fueron implementados en el SUS para, además de promover la reducción de las tasas de cesáreas, cualificar la atención obstétrica y brindar una atención humanizada, que respete la autonomía de la mujer y el proceso fisiológico del parto^(5)^. La selección adecuada de embarazos de bajo riesgo, profesionales capacitados y cualificados y el ambiente de los CPNp (una estructura que favorece el parto fisiológico, a diferencia del parto hospitalario), pueden dar como resultado partos con menos intervenciones, que no comprometan los desenlaces maternos y neonatales^(33)^.

En el presente estudio, la tasa de parto normal fue del 90,8%. Una revisión sistemática en países de altos ingresos informa que la tasa de parto normal de la atención iniciada en CPN es del 83%, mientras que en la atención iniciada en el hospital la tasa es del 61,7%^(28)^. Un estudio australiano informó que recibir atención durante el trabajo de parto en un CPN aumenta tres veces la probabilidad de tener un parto normal^(22)^. Además, una revisión integradora, que analizó 56 estudios, demostró que los partos realizados en CPNp tuvieron un número significativamente menor de intervenciones^(7)^. Por lo tanto, la elección del lugar de parto incide directamente en el tipo de parto; es decir, planificar el parto fuera del entorno hospitalario aumenta la probabilidad de parto normal y reduce las cesáreas y los partos instrumentales^(22,28)^.

La Guía de Atención del Parto Normal de Brasil y el *Intrapartum Care Guideline* del Reino Unido recomiendan el uso prudente de la oxitocina y la amniotomía^(16-17)^. El uso de oxitocina en este estudio fue incluso menor que en otros CPNp brasileños, donde la administración de oxitocina se realizó en el 27,5%-30,7% de los casos^(6,8)^. Sin embargo, la práctica de amniotomía fue mayor que la del CPNp de Río de Janeiro (1,2%)^(6)^. No fue posible comparar estos datos con estudios realizados en países de altos ingresos, ya que la oxitocina no está disponible para ser utilizada durante el trabajo de parto en los CPNp, sino solo para la prevención y el manejo de la HPP. Cuando se identifica que es necesario llevar a cabo la conducción del parto, la mujer es derivada al hospital^(17)^.

La tasa de derivación intraparto varía mucho en los estudios nacionales e internacionales (3,6%-37,4%)^(8,15,34-36)^, y es mayor en mujeres primíparas (22,5%-34,1%) que en multíparas (2,9%-12,2%)^(22,34)^. Es necesario estudiar mejor esta gran variación, sin embargo, puede estar asociada a las particularidades de cada institución, como ubicación, proximidad y convenios con el hospital de referencia, profesionales que atienden el parto, protocolos de atención y derivación, disponibilidad de recursos humanos y materiales, entre otros.

En lo que respecta a las derivaciones posparto y neonatales, hay menor discrepancia entre los estudios. Las derivaciones posparto rondan el 1,0% y las derivaciones neonatales están entre 6,1% y 6,9%^(6,35)^.

Los buenos resultados de la atención a las mujeres ingresadas para parto en *Casa Angela* son independientes de la paridad, es decir, las intervenciones y los desenlaces maternos y neonatales indican el cumplimiento de las recomendaciones para el éxito del modelo^(5,12,16,28)^. Esta conclusión se basa en estudios nacionales e internacionales que comparan a las mujeres de bajo riesgo que planearon dar a luz en Casas de Parto con las que planearon dar a luz en hospitales^(8,15,21-22)^.

En cuanto a los RN, se destacan los excelentes puntajes de Apgar, que coinciden con los encontrados en diferentes Casas de Parto brasileñas, donde el 95,2%-99,6% de los bebés obtuvieron Apgar ≥ 7 en el 1^er^ minuto y 98,9%-99,9% en el 5º^(6,8)^. Un estudio reciente realizado en Estados Unidos también observó que el 97,9% de los bebés tenía Apgar ≥ 7 en el 5.º minuto de vida^(20)^. En CPNp, los factores asociados con Apgar < 7 al 5. minuto incluyen complicaciones como distocia de hombros, prolapso del cordón, sangrado intraparto y estado fetal no tranquilizador^(8)^ y, en estudios comparativos, los puntajes de Apgar son similares a los encontrados en bebés de bajo riesgo nacidos en el hospital^(6,20)^.

Con respecto a la derivación neonatal, en el CPNp los recursos están restringidos a la atención de bajo riesgo; por lo tanto, en los casos en los que se requiere realizar una evaluación médica, exámenes complementarios o tratamientos específicos, como la fototerapia, hay que derivar al RN al hospital. Dichos motivos no se relacionan directamente con la atención del parto. Como resultado, en este estudio, la gran mayoría de los RN (77,9%) fueron trasladados por motivos no relacionados con el parto e ingresados en AC, no presentaban repercusión clínica grave y no fue necesario ingresarlos a la UTIN o UCIN.

En cuanto a los ingresos a UTIN o UCIN, se encontraron resultados similares en estudios internacionales (1,2%-2,6%), algunos no especificaban si se trataba de cuidados intensivos o intermedios^(28)^. En el presente estudio, los principales motivos de hospitalización en estas unidades de atención fueron dificultad respiratoria, infección o riesgo de infección, Apgar bajo, asfixia o hipoxia perinatal, ictericia y síndrome de aspiración de meconio.

Si bien observamos que la probabilidad de que el RN sea ingresado en una UTIN o UCIN era 5,2 veces mayor en las mujeres nulíparas, un estudio sobre CPN intra y perihospitalaria en el Reino Unido no encontró diferencias con la paridad^(37)^.

La morbilidad neonatal es una de las principales preocupaciones en la atención del parto en CPNp y destaca la importancia de cualificar a las obstetrices y enfermeras obstétricas para la evaluación y atención inmediata de los RN.

Lo mismo ocurre con la muerte neonatal. Este estudio encontró una tasa del 0,06%, mientras que la revisión sistemática de países de altos ingresos encontró 0,02%^(28)^ y el estudio estadounidense 0,03%^(20)^. Sin embargo, un estudio realizado en CPNp australianos informó una tasa del 0,3%^(36)^, cinco veces más alta que la del presente estudio.

Cabe destacar que, a pesar de las diferencias encontradas en las tasas de morbilidad y mortalidad entre los estudios sobre CPNp, estas no son diferentes de las encontradas en bebés de mujeres con bajo riesgo que dan a luz en un ambiente hospitalario^(13)^.

En *Casa Angela* solo se presentaron dos casos de mortinatos (0,06%) durante el periodo analizado por este estudio, tres veces más que los datos de la revisión sistemática de estudios de países de altos ingresos, que informaron un 0,02% de mortinatos^(28)^. Sin embargo, un estudio australiano reportó un porcentaje cuatro veces mayor, 0,24%^(22)^.

Los datos de mortalidad deben analizarse con cautela debido a la rareza del evento y al tamaño de la muestra de los estudios. La tasa de partos que ocurren en CPNp es de alrededor del 0,3% en Brasil^(1)^, lo que imposibilita calcular el tamaño de la muestra utilizando la mortalidad neonatal como desenlace primario; por lo tanto, cuando ocurre una muerte en un estudio con una muestra no calculada para ese desenlace, los datos informados pueden estar sesgados.

La mayoría de los estudios se centran en los resultados de los partos en los CPNp y no realizan un seguimiento de las mujeres y los bebés después de derivarlos al hospital. Esto afecta negativamente la calidad de los datos y puede estar relacionado con las tensiones y desafíos que aún existen en la relación entre las Casas de Parto y los hospitales de referencia.

La paridad, como predictivo de intervenciones (oxitocina y amniotomía) y de desenlaces indeseables (cesárea, fórceps, laceración de 2.º grado, episiotomía, HPP, derivación intraparto y hospitalización del RN en UTIN y UCIN), indica que la primiparidad aumenta la probabilidad de las mismas, excepto la derivación postparto. Si bien las mujeres primíparas tienen peores desenlaces que las mujeres multíparas en este CPNp, los mismos no son peores que los desenlaces que tendrían si decidieran dar a luz en el hospital^(6,8,21-22)^. La relevancia de este hallazgo indica que es necesario prestarles mayor atención a las mujeres primíparas que a las multíparas, pero también destaca la calidad y seguridad de la atención que se brinda.

El modelo de atención, la cultura local, los recursos físicos y materiales, la organización del trabajo, los recursos humanos, el liderazgo y la facilidad de cambio y adaptación son condiciones importantes para que la atención del parto se realice con menos intervenciones y un uso adecuado de tecnologías^(38-39)^. Si bien, desde el lanzamiento del Guía de Atención del Parto Normal, que les recomiendan a los profesionales de la salud informar que los CPNp son una opción segura para las mujeres de bajo riesgo, el potencial de estos establecimientos aún no se aprovecha, ya sea por la subutilización de las Casas de Parto existentes, el casi inexistente incentivo para crear nuevos CPNp, o porque no se reconoce la autonomía de las obstetrices y enfermeras obstétricas en la atención del parto. Cabe destacar que otros países también enfrentan las mismas barreras y dificultades para expandir este modelo de servicio^(40-41)^.

Este estudio concuerda con las prioridades de investigación para enfermería propuestas por la Asociación Brasileña de Enfermería, que recomienda el análisis de indicadores de atención en los CPN^(42)^.

Sin embargo, dados los buenos resultados observados en este estudio, se deberían fomentar más investigaciones sobre CPNp. Este lugar de parto, junto con el liderazgo de la atención que brindan las obstetrices y enfermeras obstétricas, es un potenciador de la lucha contra el modelo biomédico e intervencionista vigente en el país.

Cabe destacar que, desde su concepción y creación, el objetivo de dichos lugares y profesionales ha sido ofrecer y promover un modelo de atención biopsicosocial, fisiológico y respetuoso.

Recomendamos que futuros estudios incluyan los desenlaces de las derivaciones intraparto, posparto y neonatales y destaquen que el modelo de atención en el CPNp está vinculado al hospital de referencia. No informar esos resultados desconoce la existencia de esta asociación colaborativa con el hospital, que hace posible y viable la existencia de los CPNp^(39)^.

Para contribuir al avance científico, también recomendamos que se lleven a cabo estudios prospectivos, a pesar de que se conocen las dificultades y el tiempo que requieren debido al bajo número de mujeres que recibe atención en estos lugares. Entendemos que de esta manera contribuimos al conocimiento y difusión de indicadores y a la promoción del CPNp como lugar de parto seguro para la atención de mujeres con embarazos de bajo riesgo.

Las limitaciones del estudio se relacionan principalmente con el diseño transversal, con las fuentes de datos secundarias (historias clínicas y registros de internación) y posibles sesgos de información y de confusión. Algunos datos relativos a las variables que caracterizan a la mujer embarazada, además del lugar y la posición de parto, han sufrido cambios importantes a lo largo de los años. Por ese motivo, no se pudo recolectar información sobre color de piel, educación, estado civil, ingreso familiar, posición de parto y lugar del parto de toda la población del estudio, solo de la que estaba disponible en las fuentes de datos.

## Conclusión

En comparación con las mujeres multíparas, las primíparas tienen mayor probabilidad de derivación intraparto, uso de oxitocina, amniotomía, laceración perineal de 2.º grado, episiotomía, HPP, fórceps, cesárea y hospitalización del RN en UTIN o UCIN.

Sin embargo, incluso en las mujeres primíparas, las prácticas de atención en el CPNp revelan uso restringido de intervenciones (oxitocina, amniotomía, episiotomía), prevalencia de integridad perineal o traumatismo perineal leve (laceraciones de 1^er^ grado) y elección por parte de las mujeres de posiciones verticales y parto fuera de la cama. Cabe destacar que las bajas tasas de cesáreas y fórceps y de morbilidad y mortalidad materna y neonatal son compatibles con el perfil de mujeres de bajo riesgo que reciben atención en el lugar.

A su vez, contar con apoyo hospitalario es fundamental para la atención de la mujer y del RN, dado que las derivaciones maternas y neonatales, incluso con tasas bajas, son un desenlace previsto en el modelo de CPNp.

Por lo tanto, los resultados indican el parto en *Casa Angela* es seguro y que las Casas de Parto son importantes como lugar de parto en Brasil para luchar contra las altas tasas de cesárea e intervenciones innecesarias durante el parto.
